# Rad18-dependent SUMOylation of human specialized DNA polymerase eta is required to prevent under-replicated DNA

**DOI:** 10.1038/ncomms13326

**Published:** 2016-11-04

**Authors:** Emmanuelle Despras, Méghane Sittewelle, Caroline Pouvelle, Noémie Delrieu, Agnès M Cordonnier, Patricia L Kannouche

**Affiliations:** 1 Univ Paris-Sud, Laboratory Genetic stability and Oncogenesis, Equipe Labellisée La Ligue Contre Le Cancer, Villejuif 94805, France; 2 CNRS—UMR 8200, Villejuif 94805, France; 3 Gustave Roussy Cancer Campus, Villejuif 94805, France; 4 CNRS—UMR 7242, Biotechnologie et Signalisation Cellulaire, Université de Strasbourg, Ecole Supérieure de Biotechnologie, Illkirch 67412, France

## Abstract

Translesion polymerase eta (polη) was characterized for its ability to replicate ultraviolet-induced DNA lesions that stall replicative polymerases, a process promoted by Rad18-dependent PCNA mono-ubiquitination. Recent findings have shown that polη also acts at intrinsically difficult to replicate sequences. However, the molecular mechanisms that regulate its access to these loci remain elusive. Here, we uncover that polη travels with replication forks during unchallenged S phase and this requires its SUMOylation on K163. Abrogation of polη SUMOylation results in replication defects in response to mild replication stress, leading to chromosome fragments in mitosis and damage transmission to daughter cells. Rad18 plays a pivotal role, independently of its ubiquitin ligase activity, acting as a molecular bridge between polη and the PIAS1 SUMO ligase to promote polη SUMOylation. Our results provide the first evidence that SUMOylation represents a new way to target polη to replication forks, independent of the Rad18-mediated PCNA ubiquitination, thereby preventing under-replicated DNA.

## Abstract (web summary)

Translesion synthesis polymerase eta has a well characterized role in replicating past UV-induced DNA lesions and has recently been shown to act at difficult to replicate sequences. Here the authors show that its SUMOylation is required to recruit pol eta at the replication fork and to prevent under-replicated DNA.

## Graphical abstract

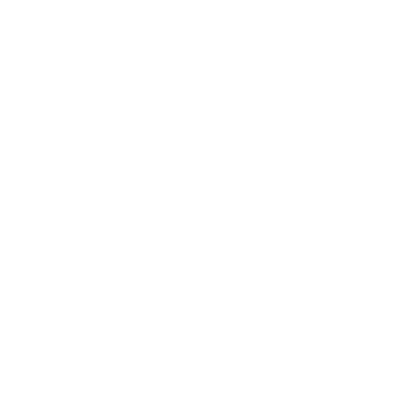

DNA polymerase eta (polη) belongs to the Y family of specialized DNA polymerases, best characterized for their capacity to replicate DNA damages that block the progression of replicative DNA polymerases, a process called translesion synthesis (TLS)^1^. Polη is particularly efficient and accurate on the most abundant damage induced by ultraviolet light, the cyclobutane thymine dimer (TT-CPD)^2^,^3^ and hereditary mutations in the *POLH* gene are responsible for the skin cancer-prone xeroderma pigmentosum variant (XPV) syndrome, highlighting the importance of TLS for genome stability. However, polη, like other TLS polymerases, is highly error-prone on undamaged templates and its access to DNA is tightly regulated through several mechanisms. For instance, mono-ubiquitination of PCNA (Ub-PCNA) by the Rad18/Rad6 complex at stalled replication forks allows specific recruitment of polη at damaged sites thanks to the cooperation of its PCNA- and ubiquitin-interacting motifs^4^,^5^,^6^. Direct interaction with Rad18 and phosphorylation also promote ultraviolet lesion bypass and cell survival^7^,^8^,^9^,^10^, whereas extraction from chromatin by the segregase valosin containing protein (VCP) and proteasomal degradation, presumably relying on ubiquitination of the TLS polymerase, were proposed to limit the extent of polη-dependent synthesis after bypass and the subsequent mutagenesis^11^,^12^,^13^.

Recently, a new function of polη at intrinsically difficult to replicate DNA loci was proposed in human cells^14^,^15^. Paragons of these loci are the common fragile sites (CFSs), which are DNA regions exquisitely prone to breakage upon mild replication stress, for instance when replicative polymerases are slowed down by a low dose of aphidicolin (APH). Incomplete replication of these loci generates DNA intermediates that can pass through mitosis, where they can be cleaved by endonucleases, generating gaps or breaks on metaphasic chromosomes^16^,^17^ or form ultra-fine bridges resolved by the Bloom pathway^18^,^19^. Stigmata of incomplete DNA replication can also be observed in the G1 daughter cells by the formation of 53BP1 nuclear bodies (53BP1 NBs), which are proposed to shield the transmitted DNA damages until repair^20^,^21^. Polη localizes at CFSs upon mild replication stress and is more efficient than the replicative polδ to replicate CFS sequences able to adopt non-B conformations *in vitro*. Moreover, APH-challenged polη-deficient cells show delayed completion of CFS replication, higher number of gaps and breaks in metaphase and accumulation of 53BP1 NBs compared with wild-type (WT) cells^14^,^15^. Polη was therefore proposed to participate in the timely completion of CFS replication, thereby preventing the persistence of under-replicated DNA in mitosis and CFS instability. As most of the knowledge on polη regulation comes from analysis of its canonical function at ultraviolet damage, it is not yet clear if this new lesion-independent function shares the same regulatory mechanisms.

Here, we show that, unexpectedly, polη travels with replication forks during unperturbed S phase and that this relies on SUMOylation of the TLS polymerase on lysine K163. Abrogation of this post-translational modification (PTM) mimics the phenotype of polη-deficient cells in response to low doses of APH, whereas it has a marginal impact after ultraviolet radiation. Rad18, independently of its ubiquitin ligase activity, promotes polη SUMOylation by facilitating its interaction with its SUMO ligase PIAS1 and is required for polη function at difficult to replicate loci. Permanently SUMOylated polη overcomes the need for Rad18 and PIAS1 in this process. Altogether, these data unravel a new way to recruit polη to replication forks, especially relevant during lesion-independent replication stress.

## Results

### polη and Rad18 travel with replication forks

**Figure 1:**
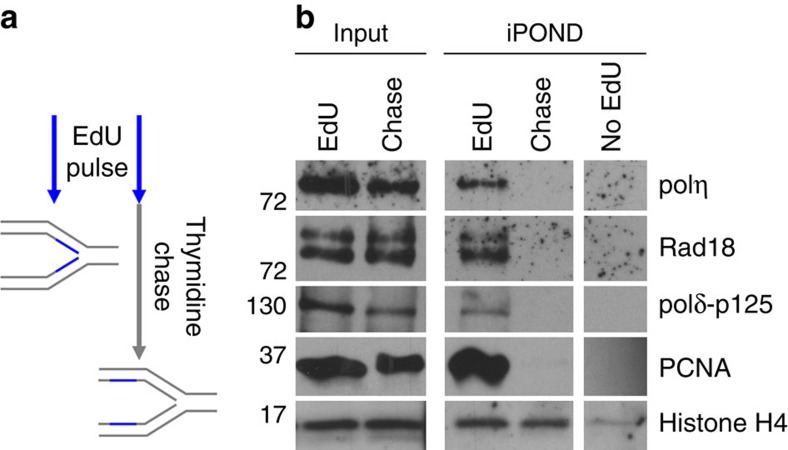
Human polη is recruited to replication forks during unchallenged S phase. (**a**) Scheme for the iPOND procedure: MRC5-V1 cells were pulse-labelled with EdU for 10 min. Cells were then crosslinked and harvested immediately or after a 1 h thymidine chase to allow replication forks moving away from the labelled DNA. Biotin was conjugated to EdU by click chemistry before cell lysis and chromatin fragmentation. EdU-containing DNA and associated proteins were purified on streptavidin beads. (**b**) Input and EdU-associated proteins (iPOND) were analysed by western blot using the indicated antibodies. No EdU: negative control processed as described in **a** but without EdU incorporation.

The discovery of polη involvement in the replication of difficult to replicate DNA loci suggests that the TLS polymerase can be recruited to replication forks in absence of DNA damage. It is known for long that overexpressed polη forms nuclear foci that co-localize with replication foci (RF) in a subset of untreated S phase cells^22^ but the localization of endogenous polη remains elusive. We therefore performed iPOND experiment (isolation of proteins on nascent DNA)^23^ in unchallenged MRC5-V1 fibroblasts. Nascent strands were pulse-labelled with the thymine analogue 5-ethynyl-2'-deoxyuridine (EdU) followed by conjugation of biotin on EdU and purification by streptavidin pull-down (Fig. 1a). Proteins associated to labelled DNA were analysed by western blot. Polη was retrieved in the sample harvested immediately after the pulse but lost in the thymidine-chased sample (Fig. 1b). This behaviour is similar to the one of known replisome components, PCNA and the catalytic subunit of the replicative polδ (p125), demonstrating that endogenous polη travels with replication forks during unperturbed S phase. Interestingly, we found that Rad18, one of its regulators, also associated with nascent DNA.

### SUMOylation on K163 drives Polη to nascent DNA

To better understand how polη is recruited to replication forks, we made the assumption that it could rely on PTMs of the polymerase. We focused on the small ubiquitin-like modifier (SUMO) pathway, as it was shown that SUMOylated proteins are enriched at replication forks^24^ and that SUMOylation was proposed to protect the *C. elegans* ortholog of polη (polh-1) from degradation during DNA damage bypass^25^.

**Figure 2:**
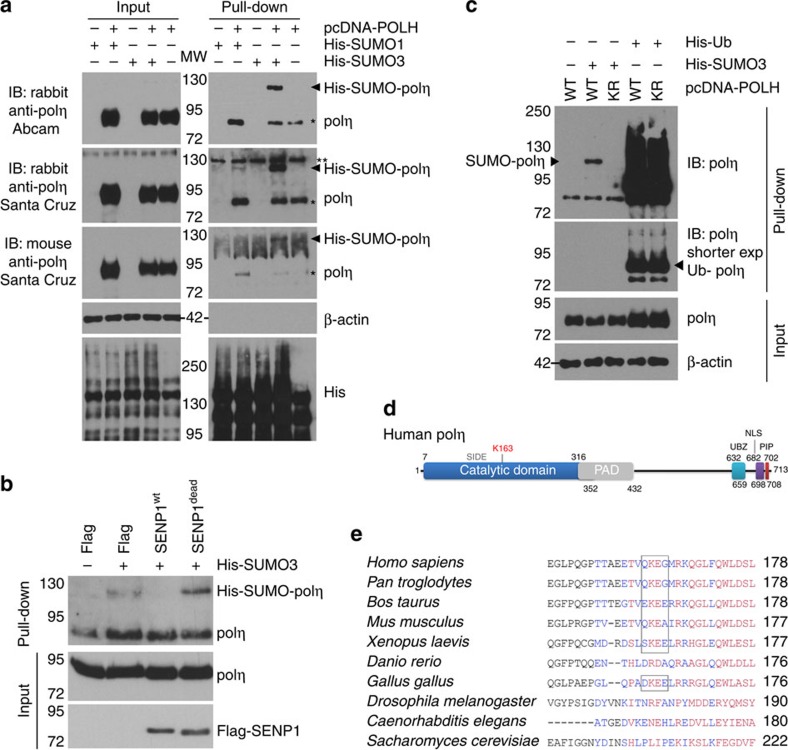
Human polη is SUMOylated *in vivo* on lysine 163. (**a**) 293FT cells were co-transfected with plasmids coding for human polη (pcDNA-POLH) and His-tagged SUMO1 or SUMO3 (His-SUMO1, His-SUMO3). Empty His and pcDNA vectors were used as controls. Cells were lysed 24 h after transfection under denaturing conditions. SUMOylated proteins were recovered on Nickel (Ni) beads. Total extracts (input) and Ni eluates (pull-down) were analysed by western blot using three different antibodies raised against polη in different species. *unspecific binding of unmodified polη to Ni beads; **unspecific band. (**b**) The impact of SENP1 SUMO protease on polη SUMOylation was analysed by denaturing Ni pull-down after co-expression of polη, His-SUMO3 and WT or catalytically dead Flag-SENP1. (**c**) 293FT cells were co-transfected with plasmids coding for WT polη or a mutant in which lysine 163 was replaced by arginine (KR) and His-tagged SUMO3 or His-tagged ubiquitin (Ub). Polη modifications were analysed as in **a**. See also Supplementary Fig. 1d for His immunoblotting. (**d**) Schematic representation of human polη. NLS, nuclear localization signal; PAD, polymerase associated domain (little finger); PIP, PCNA-interacting peptide; UBZ, ubiquitin-binding zinc finger. (**e**) Sequence alignment of polη homologues in various species. The SUMOylation site is highlighted in boxes.

Therefore, to examine if human polη is a SUMO target, 293FT cells were co-transfected with plasmids coding for WT polη (polη^WT^) and His-tagged SUMO1 or SUMO3. SUMOylated proteins were purified on nickel (Ni) beads in denaturing conditions and analysed by western blot using three different anti-polη antibodies (Fig. 2a). All the antibodies detected a slower migrating band in the pull-down, preferentially in the presence of His-SUMO3 (arrow). This band was no longer detected upon overexpression of the SUMO protease SENP1 but not of a catalytically dead SENP1 mutant (Fig. 2b), confirming that it is a SUMOylated species and suggesting that SENP1 is responsible for polη deSUMOylation. SUMO-modified polη was also detected with Flag-polη using an anti-Flag antibody (Supplementary Fig. 1a). The increase of the molecular weight of the polymerase (∼40 kDa) suggests that SUMOylated polη may contain more than one SUMO moiety. Mutation of K11 of SUMO3 to arginine (R), which prevents the formation of SUMO chains^26^, did not modify the apparent size of the modification (Supplementary Fig. 1b), showing that it is mono-SUMOylation(s).

The two Ks SUMOylated in *Caenorhabditis elegans* polh-1 are conserved in human polη; however, their mutations did not prevent its SUMOylation (Supplementary Fig. 1c). To identify the SUMO acceptor site(s), we performed *in silico* analysis with three SUMOylation site-prediction software programs (SUMOplot http://www.abgent.com/sumoplot, seeSUMO^27^ and SUMOsp^28^) and tested K to R mutants of the common predicted sites. We identified K163 as the SUMO acceptor site using denaturing Ni pull-down (Fig. 2c and Supplementary Fig. 1d). To confirm our findings, we co-expressed green fluorescent protein (GFP)-polη^WT^ or GFP-polη^K163R^ with HA-SUMO2 and purified GFP-polη on GFP-trap beads followed by extensive washes in stringent denaturing conditions. A slower migrating band was detected by both anti-polη and anti-HA antibodies only with GFP-polη^WT^ (Supplementary Fig. 1e). K163R mutation did not affect polη ubiquitination (Fig. 2c and Supplementary Fig. 1d), in agreement with previous results mapping the ubiquitination sites in the C-terminus of the polymerase^29^ and suggesting that SUMOylation is not a prerequisite for polη mono-ubiquitination.

**Figure 3:**
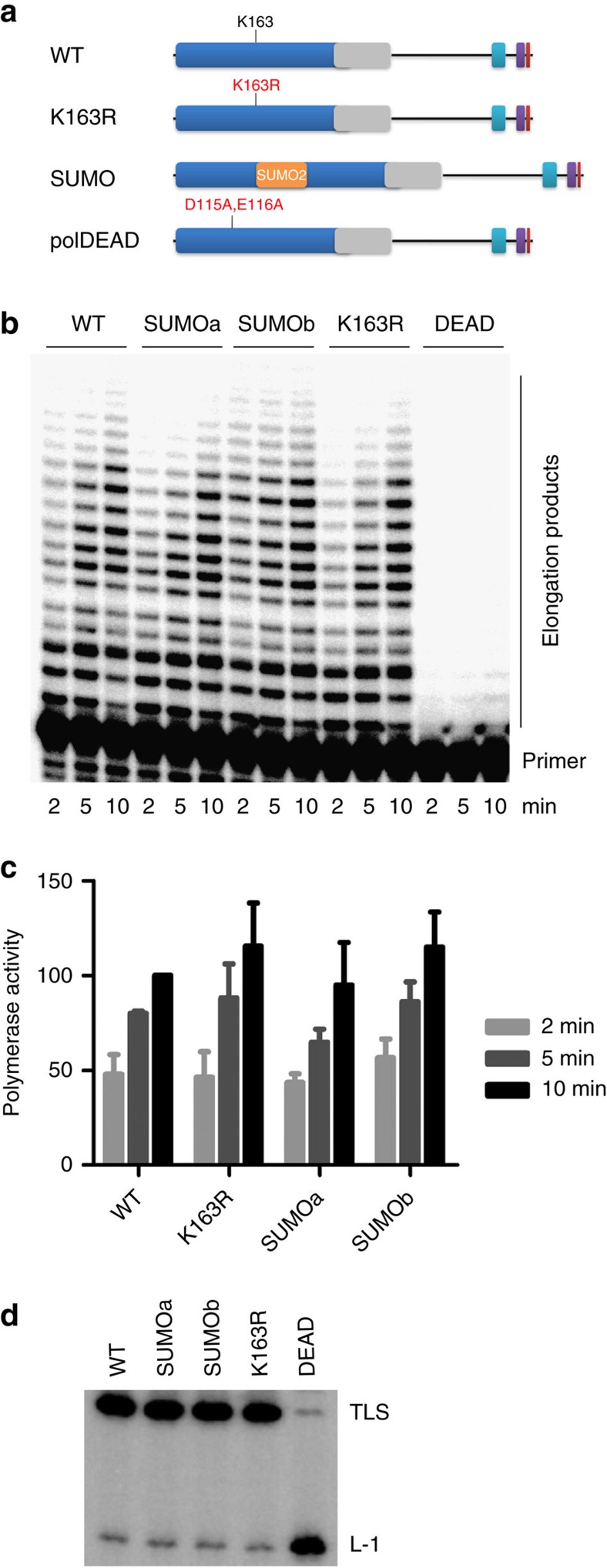
K163R and SUMO polη retain full catalytic activity *in vitro*. (**a**) Schematic representations of the polη mutants used in the study. In addition to WT and K163R polη, a constitutively SUMOylated polη (SUMO) was constructed by inserting the sequence of SUMO2 in place of K163. A track of 7 glycines (G) was added before SUMO2 to confer flexibility. The C-terminal di-G motif of SUMO2 was mutated to alanines to prevent cleavage by the SENPs. Two constructs were generated: SUMOa contains the atg of SUMO2, whereas it was mutated to G in SUMOb. A catalytically inactive polη (polDEAD) was used as a negative control. (**b**) The indicated proteins were produced in rabbit reticulocytes and their catalytic activity was analysed by primer extension on a circular single-stranded undamaged template (representative gel). (**c**) Polymerase activity is shown as a percentage of the activity measured for WT polη in 10-min reaction (mean±s.d. of three independent experiments). (**d**) For analysis of TLS efficiency, equal amounts of the *in vitro* translated proteins were introduced in an XPV cell extract. Bypass of a single TT-CPD (TLS) was assessed as described in Methods. L-1: fragment elongated up to one nucleotide before the lesion.

K163 lies in the catalytic domain of polη, in the back of the palm domain, and the SUMOylation site is conserved in most vertebrates, at the exclusion of zebrafish (Fig. 2d,e and Supplementary Fig. 1f, ref. 30). To explore if SUMOylation can impact on the intrinsic activity of the polymerase, we generated, in addition to polη^K163R^, a mimetic of constitutively SUMOylated polη (polη^SUMO^) by inserting the sequence of SUMO2 in place of K163 (Fig. 3a and Methods). Polη^K163R^ and polη^SUMO^ were fully competent for replication of undamaged DNA and for TT-CPD bypass *in vitro* (Fig. 3b–d). Hence, both non-SUMOylable and constitutively SUMOylated polη retained full intrinsic polymerase activity and the introduced mutations do not alter the conformation of polη catalytic site.

**Figure 4:**
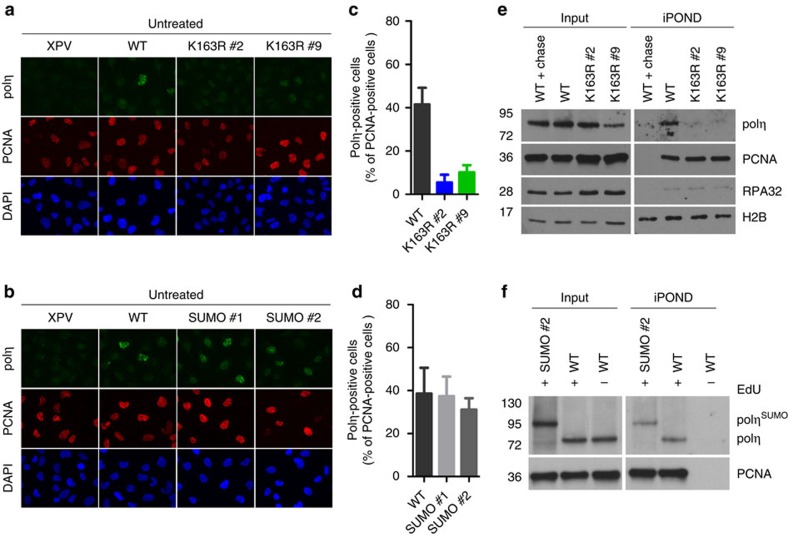
SUMOylation of polη is crucial for its recruitment at replication forks during unperturbed S phase. (**a**,**b**) Untreated XPV cells stably expressing polη^WT^, polη^K163R^ or polη^SUMO^ were fixed after extraction of soluble proteins and immunostained with polη and PCNA antibodies. Parental XPV cells were used as a negative control for polη staining. Representative images are shown (magnification × 63). (**c**,**d**) The proportion of PCNA-positive cells presenting polη foci was assessed in three independent experiments (mean±s.d.). At least 200 cells were counted per condition and experiment. (**e**,**f**) iPOND experiments were performed as described in Fig. 1.

We next investigated the biological significance of polη SUMOylation by establishing XPV cells stably expressing polη^K163R^ or polη^SUMO^. Both mutants localized in the nucleus and we confirmed that polη^K163R^ is not SUMOylated in these conditions (Supplementary Fig. 2). We first examined by immunofluorescence the capacity of these mutants to form spontaneous foci. Cells were pre-extracted with a detergent before fixation to unravel the fraction of polη associated to nuclear structures and PCNA was used as a marker of RF^6^,^22^. Only 10% of polη^K163R^ S phase cells presented spontaneous polη foci, compared with 40% for polη^WT^ (Fig. 4a,c). Moreover, polη^K163R^ foci were fainter although total polη^K163R^ amounts were similar to that of polη^WT^ (Supplementary Figs 2 and 4a). In contrast, polη^SUMO^ was fully proficient in spontaneous focus formation (Fig. 4b,d).

To determine if the impairment of spontaneous focus formation reflects a defect of polη^K163R^ recruitment to replication forks, we performed iPOND in our stable cell lines. Whereas both polη^WT^ and polη^SUMO^ were found at replication forks, the K163R mutation abolished polη recruitment to nascent DNA (Fig. 4e,f). *In situ* proximity ligation assay (PLA) between polη and neo-synthesized DNA confirmed this finding (Supplementary Fig. 3). Importantly, although MRC5-V1 and polη^WT^ cells showed specific PLA signals compared with XPV cells, only background amplification was detected in polη^K163R^ cells, despite a 3–4-fold overexpression compared with endogenous polη level (Supplementary Fig. 2a). Altogether, these results strongly suggest that polη association with the replication machinery in unchallenged conditions required its SUMOylation on K163.

### SUMO-polη increases after replication stress

**Figure 5:**
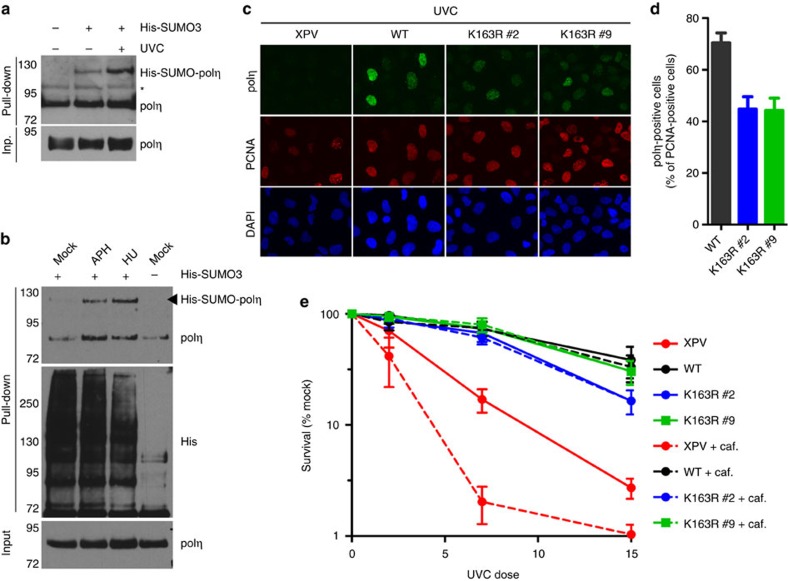
SUMOylation of polη increases after replication stress while has a minor impact on polη function at ultraviolet-induced DNA lesions. (**a**,**b**) XPV cells stably expressing polη^WT^ were transfected with His-SUMO3. 24 h after transfection, cells were irradiated at 20 J m^−2^ and incubated for 6 h (**a**) or treated with 0.3 μM APH or 0.2 mM hydroxyurea (HU) for 24 h (**b**) before performing denaturing Ni pull-down. Bound material was analysed by immunoblotting using the indicated antibodies. *unspecific band. (**c**,**d**) XPV cells stably expressing polη^WT^ or polη^K163R^ were irradiated with ultraviolet-C (20 J m^−2^), incubated for 6 h and processed as described in Fig. 4a–d. (**e**) XPV, polη^WT^ and polη^K163R^ cells were irradiated with ultraviolet-C at the indicated doses and incubated for 72 h in medium supplemented or not with 0.38 mM caffeine. Living cells were counted in the presence of trypan blue. Data are expressed as the percentage of living cells compared with mock-treated cells (mean±s.d. of four independent experiments).

If SUMOylation on K163 constitutes a means to recruit polη to replication forks, one obvious question is how this PTM impacts on the canonical and non-canonical functions of polη during S phase. To answer this, we first determined the consequence of replication stress on polη SUMOylation. Polη^WT^ cells were transfected with His-SUMO3 and exposed to ultraviolet-C or to low doses of replication inhibitors APH and hydroxyurea. Denaturing Ni pull-downs showed that SUMOylated polη was readily observed in mock-treated cells and that its level increased after both DNA lesion-dependent or -independent replication stress (Fig. 5a,b).

As previously observed^6^,^22^, ultraviolet-C exposure led to the accumulation of polη^WT^ in RF (Fig. 5c,d). Polη^K163R^ was also able to relocalize to RF after ultraviolet-C, although in only 45% of S-phase cells versus 70% for polη^WT^ and with a fainter staining (Fig. 5c,d and Supplementary Fig. 4a,b). In spite of this defect, polη^K163R^ was able to complement the ultraviolet sensitivity of XPV cells and polη^K163R^ cells were not further sensitized by addition of a low concentration of caffeine, a characteristic feature of XPV cells used for diagnostic^31^ (Fig. 5e). Accordingly, polη^K163R^ prevented the accumulation of single-strand DNA during replication of ultraviolet-damaged DNA^32^ as efficiently as polη^WT^ (Supplementary Fig. 5). However, polη^K163R^ cells were significantly more sensitive than polη^WT^ cells at a higher ultraviolet-C dose (Supplementary Fig. 4c), suggesting that polη SUMOylation can contribute to its recruitment at ultraviolet-stalled forks. Interestingly, polη^SUMO^ cells displayed WT sensitivity to ultraviolet-C. However, we observed a slight but reproducible sensitization by caffeine at a high ultraviolet-C dose (Supplementary Fig. 4d–f), suggesting that deSUMOylation is required to ensure efficient polη function at ultraviolet-damaged sites.

### Abrogation of polη SUMOylation leads to under-replicated DNA

**Figure 6:**
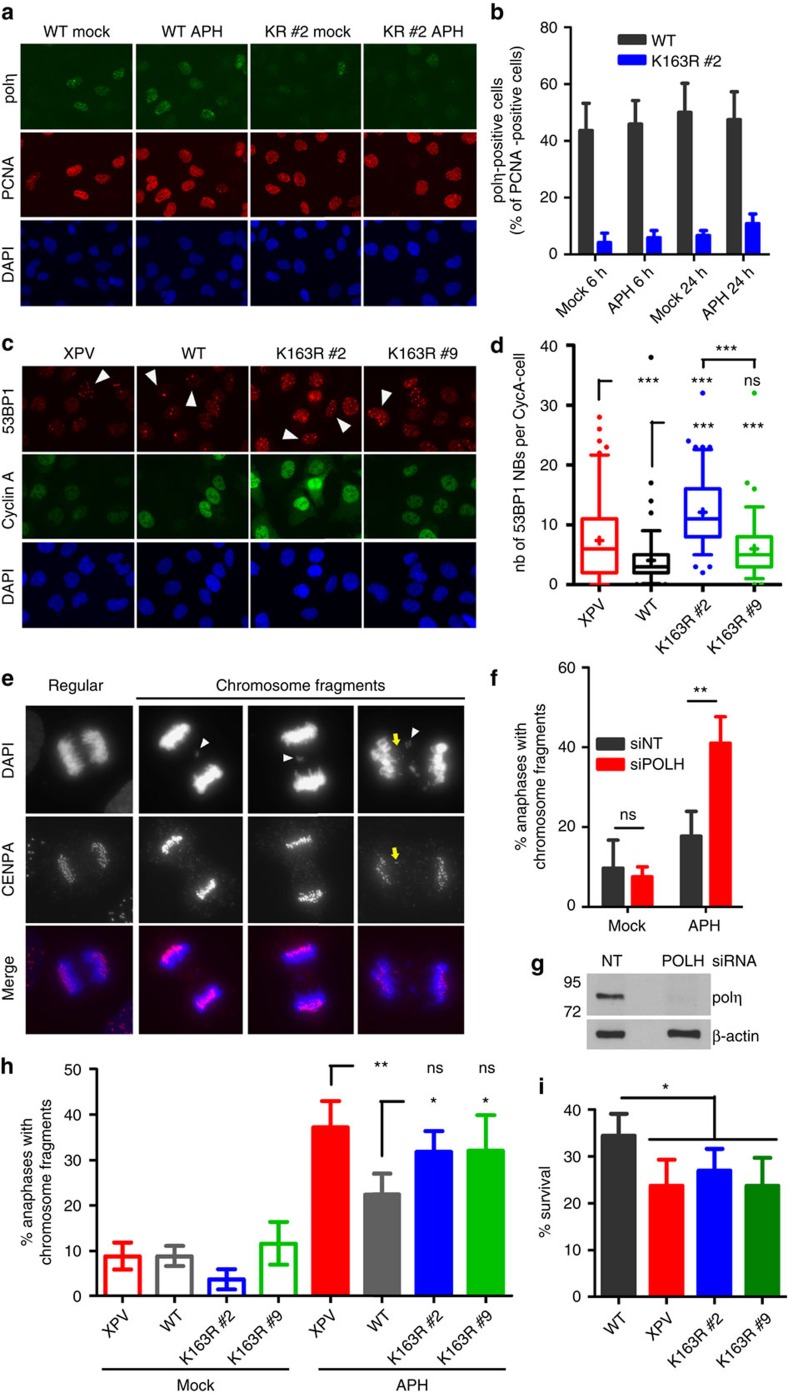
Abrogation of polη SUMOylation leads to replication defects in response to mild replication stress. (**a**,**b**) XPV cells stably expressing polη^WT^ or polη^K163R^ were treated with 0.3 μM APH for 6 or 24 h and processed as described in Fig. 4a–d. The a panel shows representative images after 24 h of APH. (**c**) XPV, polη^WT^ and polη^K163R^ cells were treated with 0.3 μM APH for 26 h before fixation and were immunostained with 53BP1 and cyclin A antibodies. Representative images are shown (magnification × 63). (**d**) The number of 53BP1 nuclear bodies (53BP1 NBs) was assessed in at least 100 cyclin A-negative cells (G1 cells, white arrows). Experiment was repeated three times, giving similar results. The distribution of 53BP1 NBs in G1 for one experiment is shown in a box-plot with 5–95 percentile whiskers (see also Supplementary Fig. 6b). ns: not significant; ***P<0.001 (Mann–Whitney test). (**e**) MRC5-V1 cells were transfected with a siRNA directed against polη mRNA (siPOLH) or a non-targeting control (siNT) 48 h before exposure to 0.15 μM APH for 24 h. Cells were fixed and centromeres were detected by immunostaining of CENPA. DNA was visualized using DAPI. Representative images of a regular anaphase and anaphases presenting lagging chromosome fragments are shown (magnification × 100). Most of the fragments lack CENPA staining (white arrows). CENPA can occasionally be found in lagging fragments (yellow arrow). (**f**) The percentage of anaphases presenting chromosome fragments was assessed in four independent experiments (mean±s.d., *n*=50 for each experiment). (**g**) Western blot confirming the efficiency of polη depletion. (**h**) The proportion of aberrant anaphases was also assessed in XPV cells and XPV cells stably expressing WT or K163R polη 24 h after treatment with 0.3 μM APH (mean±s.d. of five independent experiments, *n*=50 for each experiment). (**i**) XPV, polη^WT^ and polη^K163R^ cells were treated with 0.3 μM APH for 72 h and surviving fraction was expressed as a percentage of mock-treated cells (mean±s.d. of four independent experiments). ns: not significant, *P<0.05, **P<0.01 (*t*-test).

In contrast to what was observed after ultraviolet, XPV and polη^K163R^ cells treated with a low dose of APH experienced similar replication problems, as evidenced by increased recruitment of RPA32 on chromatin compared with polη^WT^ cells (Supplementary Fig. 5). Moreover, APH did not increase polη^K163R^ association to RF (Fig. 6a,b and Supplementary Fig. 6a), indicating that mild replicative stress is not sufficient *per se* to restore polη^K163R^ focus formation.

We then assumed that SUMOylation of polη on K163 could be required to prevent the persistence of under-replicated DNA at difficult to replicate loci^14^,^15^. To test this hypothesis, we first analysed the transmission of DNA damage to the daughter cells in the next G1 phase following APH exposure. XPV cells displayed a higher number of 53BP1 NBs per G1 cell compared with polη^WT^ cells, as already shown^14^. This defect was not corrected by the stable expression of polη^K163R^ (Fig. 6c,d and Supplementary Fig. 6b). Interestingly, we found that polη depletion in MRC5-V1 cells lead to segregation defects upon APH exposure with an increased number of anaphases presenting lagging chromosome fragments, in majority devoid of centromeric protein CENPA (Fig. 6e–g). This phenotype, evocative of increased chromosomal breaks, was also observed in XPV cells compared with polη^WT^ cells and was not rescued by polη^K163R^ expression (Fig. 6h). Moreover, polη^K163R^ and XPV cells showed similar slightly higher sensitivity to a low dose of APH (Fig. 6i). In agreement with its efficient recruitment to replication forks, polη^SUMO^ complemented the APH-induced defects of XPV cells (Supplementary Fig. 6c). However, this effect was only partial in the clone expressing the highest polη^SUMO^ level (#2), suggesting that overexpressed permanently SUMOylated polη may interfere with the correct processing of some replication intermediates.

We then asked whether polη SUMOylation impairment could affect genetic stability without impacting on cell survival after ultraviolet. We showed that polη deficiency led to a dose-dependent increase of anaphases with chromosome fragments after ultraviolet irradiation (Supplementary Fig. 7). However, both polη^K163R^ and polη^SUMO^ were able to correct this phenotype, again arguing for a minor role of polη SUMOylation at ultraviolet-induced DNA lesions.

Altogether, these results indicate that SUMOylation on K163 is required for polη recruitment at replication forks and its subsequent involvement in preventing persistence of under-replicated DNA at difficult to replicate loci. Abrogation of this PTM mimics polη deficiency in this specific function.

### Polη recruitment on nascent DNA requires PIAS1 SUMO ligase

**Figure 7:**
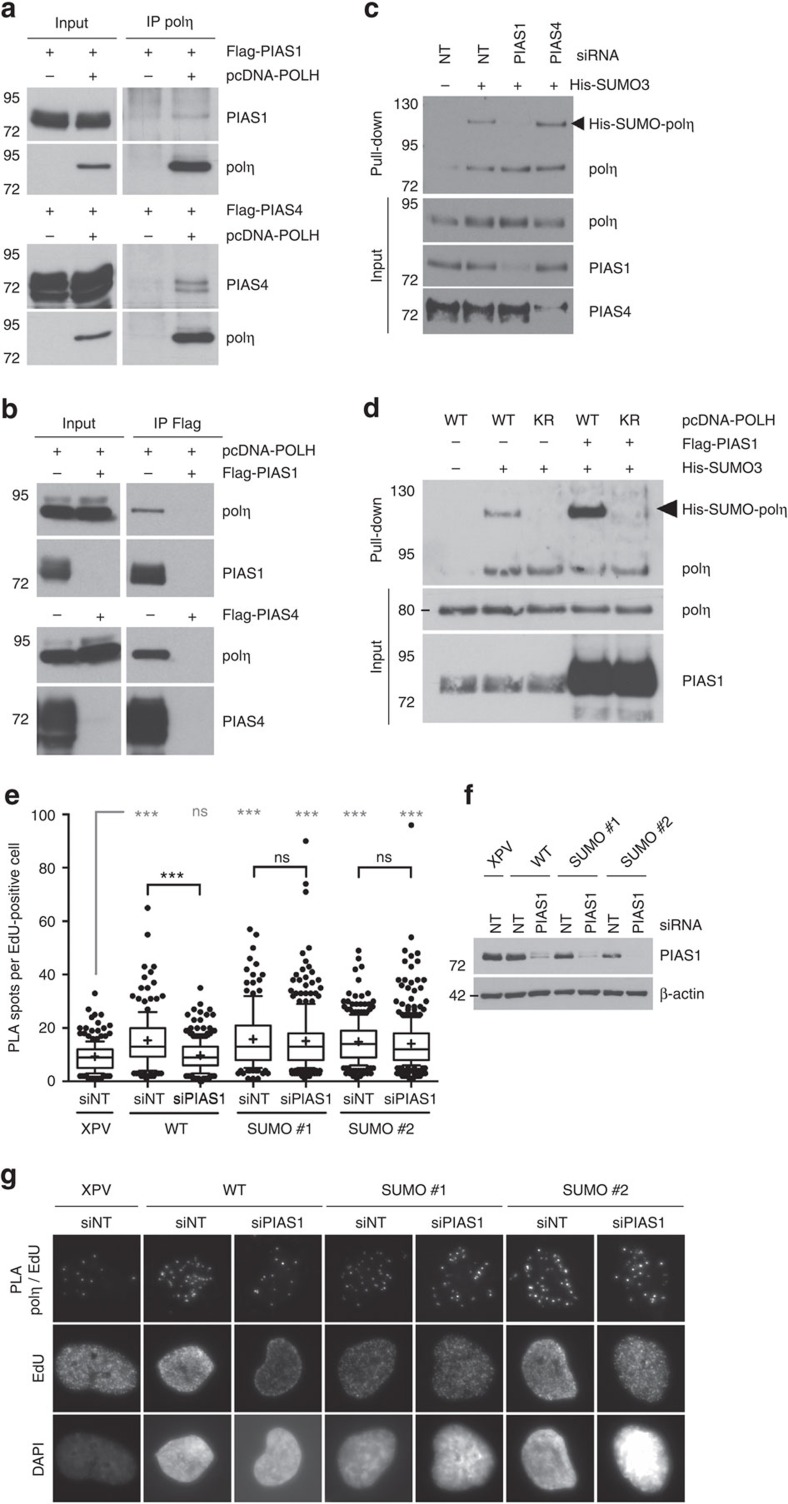
Polη is SUMOylated in a PIAS1-dependent manner. (**a**) 293FT cells were transfected with pcDNA-POLH and Flag-PIAS1 or Flag-PIAS4 and co-immunoprecipitations were performed using an anti-polη antibody. (**b**) Reversed immunoprecipitations were performed with an anti-Flag antibody. (**c**) 293FT cells were transfected with the indicated siRNAs 24 h before co-transfection of plasmids expressing polη and His-SUMO3. Denaturing Ni pull-down was carried out as described in Fig. 2. (**d**) WT or K163R (KR) polη was co-expressed in 293FT cells with His-SUMO3 and Flag or Flag-PIAS1 and cells were processed as in **c**. (**e**) XPV, polη^WT^ and polη^SUMO^ cells were transfected with non-targeting or PIAS1 siRNAs. Nascent DNA was pulse-labelled with EdU for 5 min and cells were pre-extracted and fixed. Biotin was conjugated to EdU by click chemistry in order to perform an *in situ* PLA between polη and EdU-biotin. EdU-biotin was then counterstained using a fluorescent secondary antibody. The distribution of the number of PLA spots per EdU-positive cells was assessed in two independent experiments. One representative experiment is shown in box-plot with 10–90 percentile whiskers (*n*>150, ns: not significant, ***P<0.001, Mann–Whitney test). (**f**) Western blot showing the efficiency of PIAS1 depletion. (**g**) Representative images of the PLA experiment quantified in (**e**) (magnification × 63).

To have a deeper insight into the regulation of polη SUMOylation, we then aimed to identify the SUMO ligase responsible for this modification. In *C. elegans*, polh-1 is SUMOylated by GEI-17 (ref. 25), which belongs to the PIAS family of E3 SUMO ligases that counts four members in human cells (PIAS1–4). Although the SUMOylation sites are not conserved from worm to human, we asked whether the E3 SUMO ligase of human polη could belong to this family. We showed that polη co-immunoprecipitated with both PIAS1 and PIAS4 (Fig. 7a,b), two SUMO ligases already involved in the DNA damage response^33^,^34^,^35^. However, only PIAS1 depletion impaired polη SUMOylation (Fig. 7c and Supplementary Fig. 8a). Conversely, PIAS1 overexpression enhanced polη SUMOylation in a K163-dependent manner (Fig. 7d). These results indicate that PIAS1 is the E3 SUMO ligase of human polη on K163.

PLA between polη and EdU showed that depletion of PIAS1 impaired the proximity of polη with newly synthesized DNA in both polη^WT^ and MRC5-V1 cells but had no significant impact on the recruitment of polη^SUMO^ (Fig. 7e–g and Supplementary Fig. 8b). Hence, recruitment of polη to nascent strands requires PIAS1-mediated SUMOylation of the polymerase and all the above data strongly suggest that this modification occurred on K163.

### Rad18 promotes PIAS1-mediated polη SUMOylation

**Figure 8:**
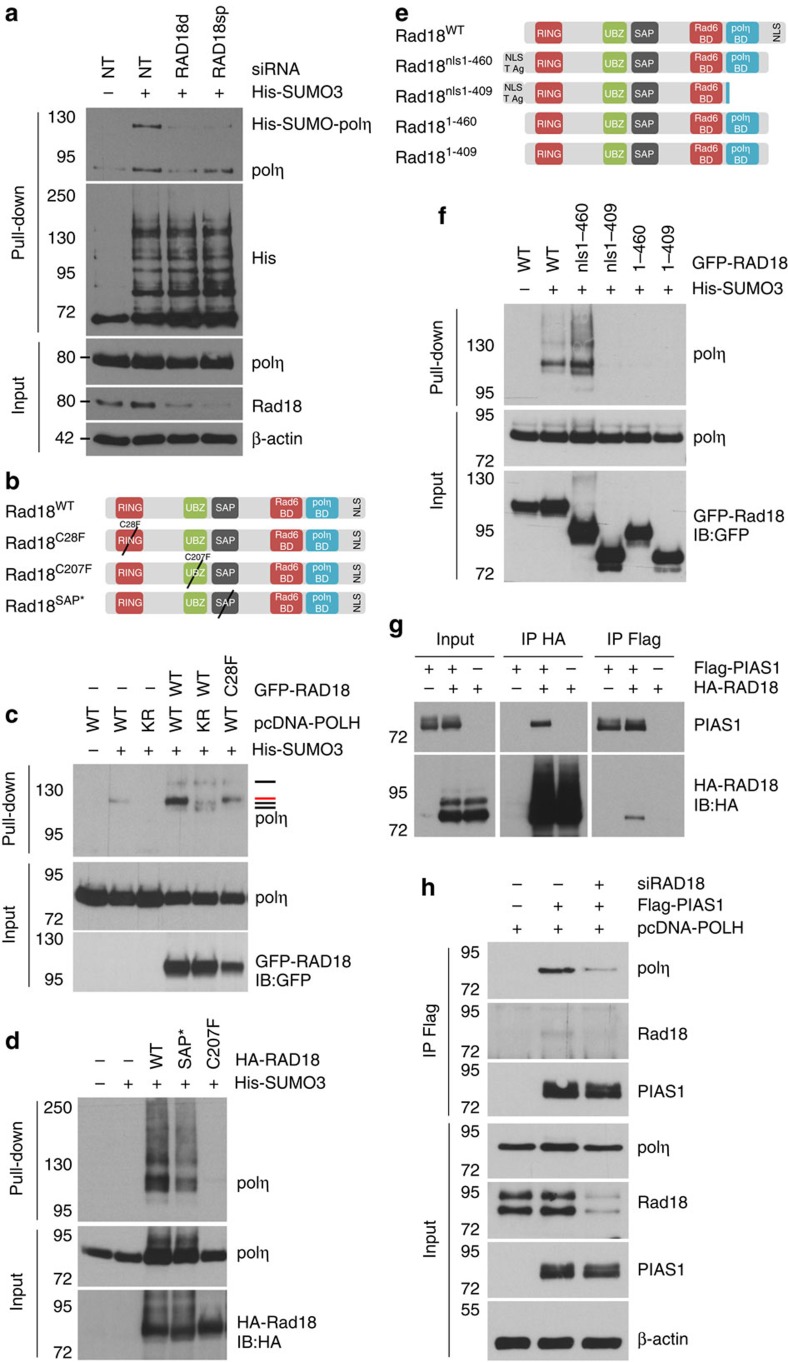
Rad18 facilitates polη SUMOylation by promoting polη interaction with PIAS1 SUMO ligase. (**a**) 293FT cells were depleted of Rad18 using either a single duplex (RAD18d) or a pool of four sequences (RAD18sp) 24 h before co-transfection of plasmids expressing polη and His-SUMO3. Polη SUMOylation was analysed as in Fig. 2. (**b**) Schematic representation of the point mutations of Rad18 used in the study. RING, really interesting new gene domain conferring ubiquitin ligase activity (mutated in Rad18^C28F^); UBZ, ubiquitin-binding zinc finger (mutated in Rad18^C207F^); SAP, SAF-A/B, Acinus and PIAS domain (mutated in Rad18^SAP*^ G269A,K271A); Rad6 BD, E2-conjugating enzyme Rad6-binding domain; polη BD: polη-binding domain; NLS, nuclear localization signal. (**c**,**d**) SUMOylation of polη was assessed after overexpression of WT Rad18 or the mutants depicted in **b**. Red line: SUMOylation on K163, black lines: K163-independent SUMOylation events. (**e**) 293FT cells were transfected with pcDNA-POLH, His-SUMO3 and various truncation mutants of GFP-RAD18 (upper panel). As Rad18^1-460^ and Rad18^1-409^ lack the C-terminal NLS, the NLS of the T antigen of SV40 (T Ag) was added to the N-terminus of the protein (Rad18^nls1-460^ and Rad18^nls1-409^). (**f**) The impact of these truncation mutants on polη SUMOylation was compared with the one of Rad18^WT^. (**g**) 293FT cells were co-transfected with HA-RAD18 and Flag-PIAS1 plasmids. Co-immunoprecipitations were performed using anti-HA or anti-Flag antibodies. (**h**) Plasmids expressing polη and Flag-PIAS1 were co-transfected in mock- or Rad18-depleted 293FT cells. The interaction between polη and PIAS1 was analysed by immunoprecipitation using an anti-Flag antibody.

Given that polη interacts constitutively with Rad18 (ref. 8), that both proteins travel with replication forks (Fig. 1b) and that depletion of Rad18 impaired polη recruitment to nascent DNA (Supplementary Fig. 8b), we investigated if Rad18 is involved in polη SUMOylation. Depletion of Rad18 using different specific siRNAs strongly impaired polη SUMOylation (Fig. 8a), indicating that Rad18 facilitates this PTM. To determine which functional domains of Rad18 act in this pathway, we analysed the impact of overexpression of various Rad18 mutants on polη SUMOylation (Fig. 8b–d). Rad18^WT^ promoted both K163-dependent and -independent SUMOylation events (see the red and black lines, respectively, in Fig. 8c). Interestingly, this was independent of its ubiquitin ligase activity (Rad18^C28F^) or its SAF-A/B, Acinus and PIAS (SAP) domain (Rad18^SAP*^), but depends on its ubiquitin-binding zinc finger (UBZ) motif (Rad18^C207F^). Noteworthy, K163-independent polη SUMOylation was markedly increased upon proteasome inhibition (Supplementary Fig. 9a), suggesting that other SUMOylation events may drive polη to degradation.

Rad18 directly interacts with the last 158 aa of polη via its polη-binding domain (BD), which was mapped between amino acids (aa) 401 and 445 (ref. 8). To determine if this direct interaction is required for polη SUMOylation, we first used a C-terminally truncated polη (polη^1-642^) and showed that it was impaired in both SUMOylation and association to Rad18 (Supplementary Fig. 9b). We next generated Rad18 truncation mutants lacking the polη BD (Rad18^1-409^) or the last 50 aa (Rad18^1–460^), which contain a nuclear localization signal (NLS) between aa 488 and 494 (ref. 36). In addition, the NLS of SV40 T antigen was added to the N-terminus of the protein to restore nuclear localization of these mutants (Rad18^nls1-409^ and Rad18^nls1-460^). Disruption of the polη BD abrogated polη SUMOylation, independently of the presence of a NLS (Fig. 8e,f). Rad18^nls1-460^ was able to promote polη SUMOylation as efficiently as Rad18^WT^, indicating that the last 50 aa of Rad18 were not required. Interestingly, Rad18^1-460^ was able to interact with polη (Supplementary Fig. 9c) but failed to promote its SUMOylation, indicating that the nuclear localization of Rad18 is important. Altogether, these results point out that direct interaction between polη and Rad18 is essential to promote polη SUMOylation in the nucleus, in agreement with the known localization of PIAS1 (ref. 37).

As a matter of fact, we showed that Rad18 interacted with PIAS1 (Fig. 8g). This required a functional NLS but was independent of Rad18 association with polη (Supplementary Fig. 9d). In contrast, depletion of Rad18 weakened the interaction between polη and PIAS1 (Fig. 8h), indicating that Rad18 may target polη to PIAS1 and/or bridge the two proteins together to allow efficient polη SUMOylation. Interestingly, polη^SUMO^ overcame the need for Rad18 for its recruitment on nascent DNA (Supplementary Fig. 9e). Altogether, our data show that direct interaction between polη and Rad18 promotes polη SUMOylation and polη recruitment to nascent DNA, independently of Rad18-mediated PCNA ubiquitination.

### SUMO-polη and Rad18 act in the same pathway after APH

**Figure 9:**
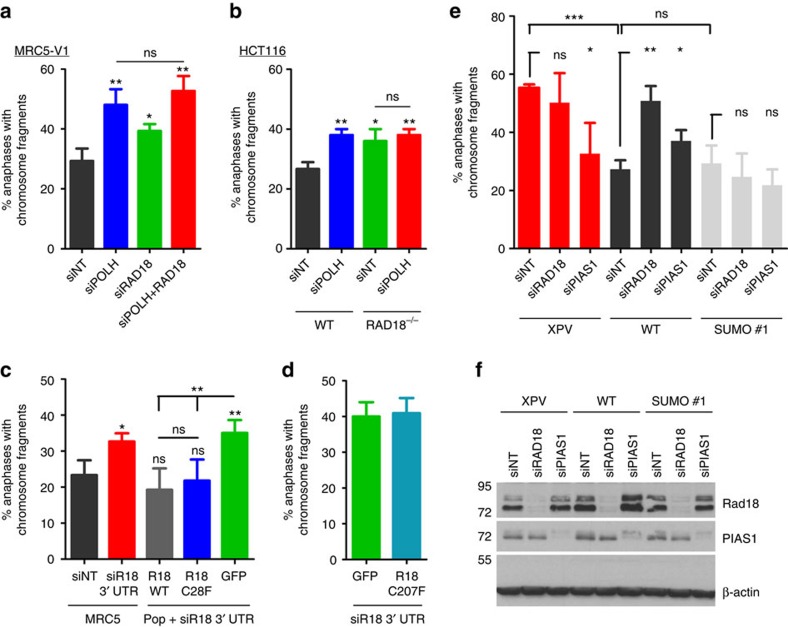
Polη and Rad18 act in the same pathway in response to replication stress and this requires Rad18 UBZ but not its ubiquitin ligase activity. (**a**) MRC5-V1 cells were transfected with siRNAs directed against polη (siPOLH) and/or Rad18 (siRAD18) mRNAs 48 h before exposure to 0.15 μM APH for 24 h. The percentage of anaphases with chromosome fragments was scored (mean±s.d. of three independent experiments, *n*=50 for each experiment). See Supplementary Fig. 10a for siRNA efficiency. (**b**) The percentage of anaphases with chromosome fragments was scored in polη-depleted *WT* or *RAD18*^−*/*−^ HCT116 cells 24 h after 0.15 μM APH (mean±s.d. of three independent experiments, *n*=50 for each experiment). See Supplementary Fig. 10c for siRNA efficiency. (**c**,**d**) MRC5-V1 cell populations expressing GFP, GFP-Rad18^WT^, GFP-Rad18^C28F^ or GFP-Rad18^C207F^ were depleted for endogenous Rad18 using a siRNA directed against the 3′-UTR of the mRNA (siR18 3′-UTR). Cells were then treated for 24 h with 0.15 μM APH and anaphases were analysed in GFP-positive cells. Data are the mean±s.d. of four (**c**) or three (**d**) independent experiments. See Supplementary Fig. 10d for siRNA efficiency. (**e**) The impact of Rad18 or PIAS1 depletion on the chromosome fragments in anaphase was determined in XPV, polη^WT^ and polη^SUMO^ cells 24 h after 0.3 μM APH. Data are the mean±s.d. of three independent experiments (*n*=50 for each experiment). ns: not significant, *P<0.05, **P<0.01, ***P<0.001 (*t*-test). (**f**) Western blot showing the efficiency of the siRNAs used in **e**.

We next showed that depletion of Rad18 increased the number of anaphases with chromosome fragments (Fig. 9a and Supplementary Fig. 10a) and the number of 53BP1 NBs in the next G1 (Supplementary Fig. 10b) after APH, in a similar manner than polη depletion. Simultaneous depletion of the two proteins did not further aggravate these defects. We confirmed this in HCT116 cells, where depletion of polη in WT cells increased the level of chromosomal fragmentation after APH to the one observed in mock- or polη-depleted *RAD18*^*−/−*^ cells (Fig. 9b and Supplementary Fig. 10c). Altogether, these data suggest an epistatic relationship between polη and Rad18 in response to mild replication stress.

We then generated cell populations expressing WT or mutated Rad18 fused to GFP. Endogenous Rad18 was depleted using a siRNA directed against the 3′-untranslated region (3′-UTR) of the mRNA (siR18 3′-UTR) and cells were treated with a low dose of APH for 24 h before scoring anaphases with chromosome fragments in GFP-positive cells. Interestingly, both Rad18^WT^ and ubiquitin ligase deficient Rad18^C28F^ were able to rescue the segregation defects observed in endogenous Rad18-depleted cells (Fig. 9c and Supplementary Fig. 10d). This suggests that ubiquitination of PCNA by Rad18 is not required in response to mild replicative stress, unlike what was previously observed after ultraviolet^38^,^39^. In agreement with that, depletion of polη in cells expressing a non-ubiquitinable PCNA mutant (PCNA^K164R^) led to increased chromosome fragmentation after APH (Supplementary Fig. 10e). In contrast, analysis of cells expressing Rad18^C207F^ showed that integrity of the UBZ motif is critical for this pathway (Fig. 9d and Supplementary Fig. 10d). These phenotypes correlate with the impact of the really interesting new gene (RING) and UBZ motifs on polη SUMOylation.

Finally, we showed that depletion of Rad18, and to a lesser extent of PIAS1, increased the number of anaphases with chromosome fragments after APH in polη^WT^ but not in polη^SUMO^ expressing cells, which demonstrates that constitutively SUMOylated polη overcomes the need for Rad18 and PIAS1 to act during mild replication stress (Fig. 9e,f). Interestingly, PIAS1 depletion significantly decreased the APH-induced mitotic defects in XPV cells, suggesting that PIAS1 may also be involved in the formation or processing of these fragments when the activity of polη is compromised.

We propose that Rad18 promotes polη SUMOylation by acting as a platform between the TLS polymerase and its SUMO ligase PIAS1, allowing polη recruitment to replication forks and prevention of under-replicating DNA in response to mild replication stress.

## Discussion

The regulation of polη access to replicating damaged DNA has been under close scrutiny since its discovery, with two underlying issues: (i) how is polη recruited to damaged sites, where its TLS activity is required, and (ii) how is TLS restricted to avoid mutagenesis on undamaged DNA? The recent discovery that polη also acts at intrinsically difficult to replicate loci^14^,^15^ modifies the way of apprehending TLS polymerase transactions on DNA. In this study, we uncovered a new mechanism, involving the SUMO pathway and Rad18, which regulates this non-canonical function of human polη during S phase.

**Figure 10:**
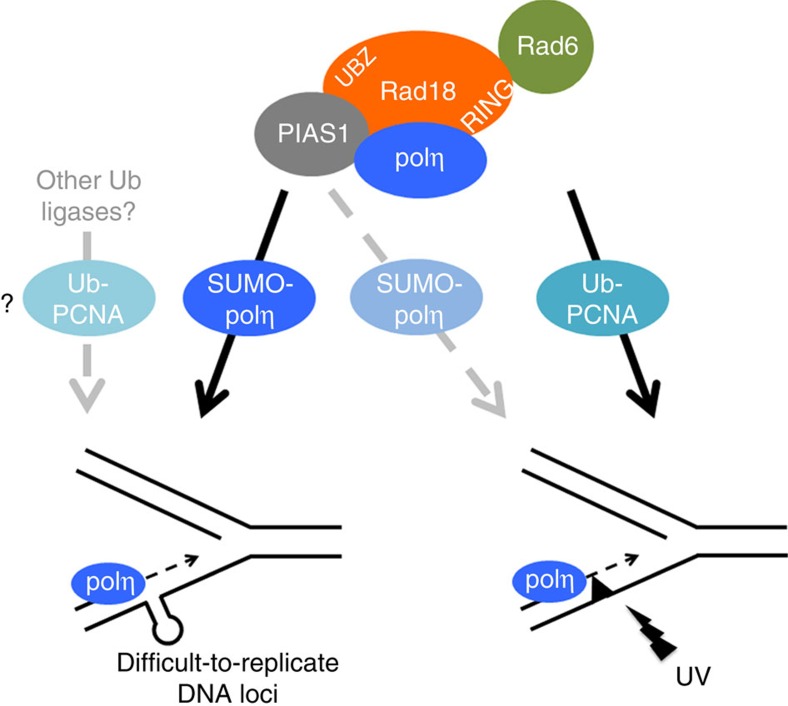
A model for the dual regulation of human polη in response to ultraviolet-C lesions and at difficult to replicate DNA loci. During unperturbed S phase or after a low dose of APH, SUMOylation of polη on K163 promotes its recruitment to replication forks to allow the timely completion of the replication of specific genomic regions presumably bearing non-B DNA structures. This PTM relies on the ternary complex formed by polη, Rad18 and the SUMO ligase PIAS1 and is independent of Rad18 ubiquitin ligase activity. Whether polη function at difficult to replicate DNA sequences also requires Rad18-independent PCNA ubiquitination remains to be established. After ultraviolet exposure, PCNA is ubiquitinated at forks stalled by photoproducts by the Rad18/Rad6 complex, which allows accumulation of polη at damaged sites, as already described. However, SUMOylation of polη on K163 may also contribute, to a minor extent, to the recruitment of the polymerase, constituting an alternative pathway in cells deficient in PCNA ubiquitination.

Our results showed that SUMOylation of polη on K163 is required for its recruitment to RF during unperturbed S phase or under low replication stress and, to a lesser extent, after ultraviolet-C irradiation. This PTM is particularly important in response to APH, preventing accumulation of ssDNA during S phase, genetic instability and cellular sensitivity. In contrast, it is largely dispensable for the efficient bypass of ultraviolet-induced lesions. Furthermore, SUMOylation of polη is promoted by direct interaction with Rad18 but independent of its ubiquitin ligase activity. We therefore propose that polη is differentially regulated in response to DNA lesions and to intrinsic replication fork barriers (Fig. 10). During unperturbed S phase or under mild replication stress, when the amounts of Ub-PCNA are low, PIAS1-mediated SUMOylation on K163 targets or retains polη in the vicinity of replication forks encountering difficult to replicate sequences, such as non-B DNA, promoting the timely completion of their replication. After ultraviolet exposure, polη relocalizes to virtually all RF, where its accumulation mainly relies on PCNA ubiquitination, as already described^4^,^6^. Our results highlight a central role for Rad18 in the regulation of polη, as it is a key factor in both processes, which rely on distinct functional domains. Indeed Rad18, in complex with the E2 ubiquitin conjugating enzyme Rad6, is responsible for the ubiquitination of PCNA, a process requiring both its RING and SAP domains, and also directly targets polη to damaged sites^8^,^40^. Here, we show that Rad18 promotes polη SUMOylation in a UBZ-dependent manner by bridging polη and its SUMO ligase PIAS1 and shares an epistatic relationship with the TLS polymerase in response to mild replication stress. Interestingly, these latter functions do not require a functional Rad18 RING domain and therefore the associated PCNA ubiquitination. However, as other ubiquitin ligases are able to ubiquitinate PCNA^41^,^42^, we cannot formally exclude that Rad18-independent ubiquitination of PCNA participates in polη function at difficult to replicate DNA loci. In particular, it would be interesting to investigate the role of the E3 ubiquitin ligase CRL4^Cdt2^, as it is responsible for a fraction of PCNA ubiquitination in untreated cells^41^. Moreover, this E3 ligase targets some PCNA-interacting proteins to degradation after ultraviolet, a mechanism required for polη focus formation^43^,^44^. As most CRL4^Cdt2^ substrates are also degraded during the course of unperturbed S phase, it is tempting to speculate that this clearance pathway operates as well during the replication of difficult to replicate loci.

We showed that the K163R mutation does not lead to a strong defect of ultraviolet-lesion bypass, as evidenced by cell survival experiments, lack of ssDNA accumulation in S phase and rescue of the mitotic defects observed in irradiated polη-deficient cells. However, in agreement with the moderate impairment of focus formation after ultraviolet, polη^K163R^ cells display increased sensitivity to high ultraviolet doses than polη^WT^ cells, suggesting that SUMOylation on K163 indeed also participates to the accumulation of polη at forks stalled by photoproducts. Recently, several studies have challenged the currently accepted model placing Ub-PCNA at the heart of TLS regulation, with data supporting Ub-PCNA independent pathway(s) for polη activation^45^,^46^,^47^. We propose that SUMOylation on K163 provides an alternative way to recruit polη at damaged sites when PCNA ubiquitination is compromised.

Hence, although canonical and non-canonical polη functions during S phase could theoretically be reconciled in a unique tolerance mechanism requiring the same stalling/recruitment/bypass steps irrespectively of the type of fork barrier, our data argue for a differential regulation of polη at DNA damage and at non-B DNA. We postulate that this may reflect requirement of different protein complexes or different impacts of these replication impediments on the structure of the replication intermediates, a subject that remains largely unexplored in human cells.

Using iPOND to retrieve the proteins associated with nascent DNA, we showed that polη and Rad18 travel with replication forks during unperturbed S phase. Noteworthy, during the preparation of this manuscript, two teams also identified Rad18 as a component of the replisome^48^,^49^. Our data on polη are, to our knowledge, the first demonstration of a TLS polymerase association with protein complexes at nascent strands in unchallenged cells. This finding was rather unexpected, given the intrinsic low fidelity of the polymerase on undamaged templates. However, our observation fits well with the emerging concept of TLS polymerases involvement in the natural course of DNA replication^50^. Moreover, polη presence in the replisome does not necessarily imply that it actively replicates DNA, a hypothesis supported by the limited number of interaction signals between polη and nascent DNA observed by PLA. Polη may be pre-recruited to rapidly cope with barriers impeding replication fork progression. Composition of the replisome varies in response to acute replication stress^23^,^48^,^51^. However, it is not yet precisely known if and how this complex is modulated in response to natural fork barriers and after mild replication stress. Therefore, it remains to be determined if polη and Rad18 are constitutive component of the replisome or if they are specifically found in the vicinity of forks dealing with the replication of problematic DNA regions like CFSs.

The current model implies that Rad18 is recruited on chromatin through the ssDNA formed at stalled forks and therefore its DNA-binding domain SAP is required for ultraviolet-induced PCNA ubiquitination and polη foci^38^,^52^. We found that polη SUMOylation and prevention of segregation defects upon APH treatment rather rely on the UBZ domain of Rad18, a motif involved in Rad18 dimerization and subnuclear localization^36^,^38^,^53^,^54^,^55^ but dispensable for PCNA ubiquitination, polη foci formation and cell survival after ultraviolet^36^,^54^. Interestingly, the UBZ motif was shown to promote interaction of Rad18 with ubiquitinated chromatin components including histone H2A^38^,^56^. Hence, it may provide a way to recruit the Rad18/polη complex to the replisome, independently of fork stalling, and/or may target them to specific DNA regions.

Our results showed that association of polη with the replisome in unperturbed S phase required its PIAS1-mediated SUMOylation on K163. Interestingly, both PIAS1 and SUMOylated species are enriched on nascent DNA^24^,^48^. However, it is not yet clear if PIAS1 SUMOylates polη in the vicinity of replication forks, despite the fact that we demonstrated that polη is SUMOylated in the nucleus. As it has been reported for many other SUMO-modified proteins, the amount of SUMOylated polη is very low compared with that of the unmodified protein and only unmodified polη was detected at replication forks. SUMO conjugation can be a very transient event, yet having a prolonged impact on the target protein. The cycling model proposed to explain this apparent paradox stipulates that SUMOylation acts through cycles of conjugation/deconjugation, SUMOylation promoting an event, like the recruitment of the target to a protein complex, which can persist after SUMO clearance^57^,^58^. Based on this model, we hypothesize that highly dynamic SUMO attachment on polη K163 allows polη stable incorporation in the replisome. The factors chaperoning this process remain to be identified.

On the other hand, it is also tempting to speculate that polη SUMOylated on K163 represent the DNA elongating form of the polymerase *in vivo* and that its small amount precludes any detection by the current methods. According to the crystal structure of human polη, the K163 residue is located in the back of the palm domain, in the most flexible region of the catalytic domain^30^. Therefore, and in agreement with our *in vitro* data, it is unlikely that attachment of a SUMO moiety alters the conformation or polymerase activity of polη. However, the fact that polη^K163R^ is proficient in ultraviolet lesion bypass *in vivo* suggests that SUMOylation is not a strict requirement for polη activity. SUMOylation on K163 might then protect polη from restrictive mechanisms during DNA synthesis, in reminiscence of what is observed in the nematode after damage^25^, the excessive turn-over of polη^K163R^ at DNA synthesis sites being partly compensated by its increased affinity for Ub-PCNA after ultraviolet.

Interestingly, polη was found as a putative SUMO target by mass spectrometry analysis of cells treated with the proteasome inhibitor MG132 (ref. 59), suggesting that SUMOylation may be a prerequisite for polη degradation. We confirmed this finding using denaturing Ni pull-down and showed that the SUMOylation events up regulated by inhibition of the proteasome are independent of K163. Therefore, SUMO pathway may fulfil two opposite roles: SUMOylation on K163 promotes polη function at difficult to replicate loci, whereas multiple SUMOylations on other unidentified sites mark the polymerase for proteasomal degradation. Recently, the segregase p97/VCP, associated to its adaptator Spartan/DVC1, has been shown to extract polη from the chromatin after lesion bypass^11^,^13^. VCP mostly acts on ubiquitinated proteins, but it is now demonstrated that it can also target SUMOylated proteins^60^,^61^. Interestingly, Spartan/DVC1 has been proposed as the functional homologue of the yeast metalloprotease wss1 (ref. 62), a partner of the yeast ortholog of VCP recently shown to bear a SUMO ligase activity^63^. One possibility is therefore that Spartan/DVC1 may be responsible for the SUMOylation events leading to polη degradation. Further investigations are required to clarify this hypothesis.

In summary, we identified a novel layer of regulation of polη to prevent under-replicated DNA at difficult to replicate loci, which involves SUMO pathway and Rad18 but not Rad18-mediated ubiquitination of PCNA. We therefore propose that polη is differentially regulated in response to DNA insults or to intrinsic replication fork barriers to maintain genome stability. The protein Rad18 serves as a common regulator for these distinct pathways.

## Methods

### Cell lines

Normal (MRC5-V1) and XPV (XP30RO) SV-40 immortalized fibroblasts (kind gift from A. Lehmann and J. E. Cleaver, respectively) were maintained in Minimal Eagle Medium (MEM Glutamax; Gibco) supplemented with 10% fetal calf serum, 100 U ml^−1^ penicillin and 100 μg ml^−1^ streptomycin. MRC5-HisPCNA^K164R^ cells^64^ (kind gift from A. Lehmann) were grown in the presence of 0.8 mg ml^−1^ G418. 293FT (Invitrogen), U2OS (kind gift from V. Pennaneach) and HCT116 (kind gift from T. Shiomi) cells were maintained in DMEM (Gibco) supplemented with 10% fetal calf serum, L-glutamine, non-essential amino acids, sodium pyruvate, 100 U ml^−1^ penicillin and 100 μg ml^−1^ streptomycin. HCT116-RAD18^−/−^ cells^65^ were grown in the presence of 300 μg ml^−1^ G418 and 0.3 μg ml^−1^ puromycin. Cells were cultivated at 37 °C under 5% CO_2_. Construction of stable XP30RO cells expressing WT polη (polη^WT^) was described elsewhere^66^. XP30RO cells expressing polη^K163R^ and polη^SUMO^ were obtained by stable transfection using ExtremGene 9 (Roche), according to the manufacturer's instructions, and selection with 150 μg ml^−1^ of zeocin (Invivogen). WT and mutant cell lines were further grown in presence of 100 μg ml^−1^ zeocin. To generate populations expressing GFP-Rad18, MRC5-V1 cells were transfected with Fugene HD (Promega) and GFP-positive cells were cell sorted and further grown in medium containing 0.8 mg ml^−1^ G418 (Gibco).

### Plasmids

Polη was expressed using pcDNA.3.1.zeo-.POLH or GFP-C3-POLH plasmids. Mutation of lysine 163 to arginine to generate polη^K163R^ was done by site-directed mutagenesis according to the manufacturer's instructions (Agilent, polηK163Rs: 5′-cggcagaagagactgttcagagagaggggatgc-3′; polηK163Ras: 5′-gcatcccctctctctgaacagtctcttctgccg-3′). A mimetic of constitutively SUMOylated polη (polη^SUMO^) was obtained by gene synthesis (Life) by replacing the K163 codon with the sequence of SUMO2 flanked by seven and two glycines upstream and downstream, respectively. This sequence was then cloned in the pcDNA.3.1.zeo- vector. We mutated the C-terminal di-G motif of SUMO2 to alanine to avoid cleavage by the SENPs (SUMOa construct, polη^SUMO^G261,262As: 5′-ccaacagcagacggcagctgtctacggtggtg-3′; polη^SUMO^G261,262Aas: 5′-caccaccgtagacagctgccgtctgctgttgg-3′). The SUMOb construct bears an additional mutation of the atg of SUMO2 to generate an eight glycine upstream the SUMO2 sequence (polη^SUMO^M170Gs: 5′-tggtggtggtggtggtggtggggccgacgaa-3′: polη^SUMO^M170Gas: 5′-ttcgtcggccccaccaccaccaccaccacca-3′). The plasmids expressing the polymerase DEAD mutant (polη^polDEAD^) and GFP-polη^1-642^ were described elsewhere^22^,^67^. Rad18 was expressed using GFP-C3-RAD18 or pCMV2-HA-RAD18 plasmids. Truncation mutants were obtained by PCR and further cloned in GFP-C3, GFP-C3-SV40nls^68^ and pCMV2-HA using the XhoI/BamHI restriction sites added to the primers (forward primer: 5′-GATTACCTCGAGATGGACTCCCTGGCCGAGTCTC-3′; reverse primer for Rad18^1-460^: 5′-ATCATGGGATCCTTATGATGCTTCCCAGGCTTCCTCTTCTTC-3′; reverse primer for Rad18^1-409^: 5′-ATCATGGGATCCCTAGGAGTCCAGCTTTGATTGAGAAAAGTG-3′). All amino-acid substitutions were done by site-directed mutagenesis (Rad18^C28F^s: 5′-atgttgaaatactcgaagaaaattccacaccgcagcaaatc-3′; Rad18^C28F^as: 5′-gatttgctgcggtgtggaattttcttcgagtatttcaacat-3′; Rad18^C207F^s: 5′-aagttactaaagtggattgtcctgttttcggggttaacattc-3′; Rad18^C207F^as: 5′-gaatgttaaccccgaaaacaggacaatccactttagtaactt-3′: Rad18^SAP*^s: 5′-aaaagagcatggattatctattcaagcaaatgcacaacagctcattaaaaggcacca-3′; Rad18^SAP*^as: 5′-tggtgccttttaatgagctgttgtgcatttgcttgaatagataatccatgctctttt-3′). Plasmids expressing His-SUMO1 and His-SUMO3 were a kind gift of Anne Dejean and Mauro Modesti, respectively. Flag-mPIAS1 and Flag-PIAS4 were a gift from Ke Shuai (Addgene plasmids # 15206 and # 15208). Flag-SENP1 and Flag-SENP6 were a gift from Edward Yeh (Addgene plasmids # 17357, # 17358 and # 18065).

### Cell treatments

For ultraviolet-C irradiation (254 nm), cells were rinsed in pre-heated PBS and irradiated without any medium at a fluency of 0.65 J m^−2^ s^−1^. APH and MG132 (Sigma) stock solutions were at 3 and 4 mM, respectively, in DMSO.

### siRNAs

siRNAs purchased from Dharmacon were used to transiently downregulate the expression of *PIAS1*, *PIAS4* (smart pool), *RAD18* (siRAD18d: 5′-CAUAUUAGAUGAACUGGUAUU-3′, siRAD18sp: smart pool; siRad18 3′-UTR: 5′-GTGTGTAAGTACCGATGCAUU-3′), *PCNA* (5′-GCCGAGAUCUCAGCCAUAUTT-3′) or *POLH* (5′-GAAGUUAUGUCCAGAUCUU-3′). Unspecific siRNAs (siNT) were used as control.

### Transfections

Unless otherwise indicated, plasmids were transfected using jetPEI (Polyplus), according to the manufacturer's instructions. Cells were transfected with 30 nM of siRNAs using Interferin (Polyplus) according to the manufacturer's instructions and incubated for 48 h before treatment. In co-depletion experiments, 15 nM of each specific siRNA was used. siNT (15 nM) was added to ensure a final concentration of 30 nM when required. HCT116 cells were transfected with calcium phosphate. For analysis of SUMOylation by denaturing Ni pull-down, 293FT cells were seeded in 60 mm dishes 1 day before plasmid transfection (1 μg of pcDNA-POLH or GFP-POLH+2 μg of His or His-SUMO±1 μg of GFP-RAD18 or Flag- PIAS or Flag-SENP). Stable XP30RO-derived cell lines were seeded in 100 mm dishes and transfected with 7 μg of His or His-SUMO3 24 h before treatment. In depletion experiments, cells were transfected with siRNAs the day after seeding and further incubated 24 h before plasmid transfection. Plasmids were allowed to express for 24 h. Alternatively, co-transfection of siRNAs and plasmids were performed by calcium phosphate 48 h before harvesting. For denaturing GFP-trap, 293FT cells were transfected with 1 μg of plasmids expressing GFP, GFP-polη^WT^ or GFP-polη^K163R^ and 2 μg of HA-SUMO2. For immunoprecipitation experiments, 293FT cells were transfected with 2 μg of each of the indicated plasmids 24 h before harvesting.

### Denaturing Ni pull-down

Cells were lysed in 500 μl of urea buffer (8 M urea and 20 mM imidazole in PBS) supplemented with 20 mM N-ethylmaleimide (NEM, Sigma) at room temperature and sonicated for 15 s with 30% amplitude (Vibracell, Bioblock Scientific). Extracts were centrifuged at 16,000*g* for 10 min at 15 °C. 50 μl of supernatant was kept as input fraction and boiled for 10 min in 2 × Laemmli buffer. Samples were incubated with nickel beads (His60 Ni Superflow resin, Clontech) for 45 min at room temperature on a wheel. Beads were washed four times for 5 min in 1 ml urea buffer. Proteins were eluted by boiling for 10 min in 2 × Laemmli buffer with 30 mM EDTA and analysed by western blot.

### Denaturing GFP-trap

Purification of GFP-polη in stringent denaturing conditions was performed according to Chromotek's application note on ubiquitination of GFP-tagged proteins. Cells were lysed in GFP-trap lysis buffer (50 mM Tris-HCl pH 7.5, 150 mM NaCl, 1 mM EDTA, 0.5% Triton × 100, 20 mM NEM, antiproteases Complete EDTA-free Roche) for 20 min on ice. Samples were sonicated twice for 10 s at 29% amplitude and cleared by centrifugation for 5 min at 9,500*g* at 4 °C. Supernatants were incubated for 2 h 30 at room temperature on a wheel with 20 μl of GFP-trap agarose beads (Chromotek). Beads were washed once with GFP-trap dilution buffer (10 mM Tris-HCl pH 7.5, 150 mM NaCl, 0.5 mM EDTA, 20 mM NEM and antiproteases), three times with stringent washing buffer (8 M urea, 1% SDS in PBS) and once with 1% SDS in PBS. Bound proteins were eluted by boiling for 10 min in 2 × Laemmli buffer.

### Immunoprecipitation

Cells were lysed in NETN buffer (50 mM Tris-HCl pH 7.5, 150 mM NaCl, 1 mM EDTA, 0.5% NP40, antiproteases) for 30 min on ice and sonicated twice at 29% for 10 s. Samples were cleared by centrifugation at 9,300*g* for 5 min at 4 °C. Immunoprecipitations were performed with 1 μg of antibodies (Bethyl rabbit anti-polη #A301-230A, Sigma mouse anti-Flag-M2 #F4049 or Santa Cruz mouse anti-HA F-7 #sc-7392) for 3 h at 4 °C on a wheel followed by 1 h 30 incubation in presence of sepharose-protein A beads (GE Healthcare). Beads were extensively washed in NETN, with 300 mM NaCl for the final wash, and denatured in 2 × Laemmli.

### iPOND

The iPOND experiment was performed as described elsewhere^51^ with minor modifications. Briefly, 10^8^ cells were pulse-labelled with 10 μM EdU (Invitrogen) for 10 min. Immediately after the pulse or after a 1 h chase in fresh medium supplemented with 10 μM thymidine (Sigma), cells were crosslinked with 1% formaldehyde (Sigma) in PBS for 15 min at room temperature under gentle agitation. Crosslink was stopped by addition of 125 mM glycine for 5 min. Cells were harvested by scrapping and washed in ice-cold PBS. Pellets were permeabilized in PBS with 0.5% Triton × 100 for 30 min at room temperature. Biotin-azide (Molecular Probes) was conjugated to EdU by click chemistry for 2 h in click reaction buffer (10 mM sodium-L-ascorbate, 10 μM biotin-azide, 2 mM CuSO_4_ in PBS). Cells were lyzed in iPOND lysis buffer (10 mM Hepes-NaOH pH 7.9, 100 mM NaCl, 2 mM EDTA, 1 mM EGTA, 1 mM PMSF, 0.2% SDS, 0.1% sarkozyl, antiproteases) and sonicated on a Bioruptor device (30 cycles of 30 s on/30 s off at the highest setting). Solubilized chromatin was retrieved by centrifugation at 16,000*g* for 10 min and supernatant was further incubated overnight with magnetic streptavidin beads (Dynabeabs MyOne Streptavidine C1, Invitrogen). Beads were washed once in lysis buffer, once in 500 mM NaCl and twice in lysis buffer. Proteins were eluted by boiling in 1 × Laemmli buffer at 95 °C for 30 min.

### Western blot

For whole-cell extract preparation, cells were lysed in SDS lysis buffer (50 mM Tris pH7.5, 20 mM NaCl, 10 mM MgCl2, 0.1% SDS, anti-proteases) supplemented with benzonase for 10 min at room temperature, as previously described^32^. Proteins were quantified with Bradford assay. Proteins were separated on 8 or 15% acrylamide SDS–polyacrylamide gel electrophoresis. Membranes were blotted with antibodies directed the following proteins: β-actin (mouse AC-15, Sigma #A5441, 1/10,000), Flag (mouse M2, Sigma #F4049, 1/1,000), GFP (mouse, Roche #11814460001, 1/1,000), histone H2B (rabbit V119, Cell Signaling #8135, 1/1,000), histone H4 (mouse, Abcam #ab31830, 1/1,000), HA (mouse HA.11 16B12, Covance #MMS-101R, 1/1,000), 6x-His tag (mouse #631212, Clontech, 1/5,000), PCNA (mouse PC10, Santa Cruz #sc-56, 1/4,000), Ub-PCNA Lys164 (rabbit D5C7P, Cell Signaling #13439, 1/1,000), PIAS1 (rabbit, Epitomics #2474, 1/5,000), PIAS4 (rabbit, ProteinTech #14242-1-AP, 1/1,000), polδ-p125 (goat C-20, Santa Cruz #sc-8797, 1/1,000), polη (rabbit, Abcam #ab17725, 1/1,000; rabbit H-300, Santa Cruz #sc-5592, 1/2,000; mouse B-7, Santa Cruz #sc-17770, 1/500; rabbit, Bethyl #A301-231A, 1/1,000), Rad18 (mouse, Abcam #ab57447; rabbit, Bethyl #A301-340A, 1/2,000), RPA32 (mouse, Calbiochem #NA19L, 1/5,000). Uncropped images for the most relevant blots are shown in Supplementary Fig. 11.

### Immunofluorescence

For analysis of polη foci, cells were pre-extracted in CSK100 buffer (100 mM NaCl, 300 mM sucrose, 3 mM MgCl_2_, 10 mM Pipes pH 6.8, 1 mM EGTA, 0.2% Triton x100, antiproteases) for 5 min on ice under gentle agitation. Cells were fixed in 4% paraformaldehyde for 20 min and permeabilized in methanol at −20 °C for 10 s. Cells were incubated for 1 h at room temperature with primary antibodies (Santa Cruz H300 rabbit anti-polη 1/300+Santa Cruz PC10 mouse anti-PCNA 1/500) diluted in IF buffer (3% BSA, 0.5% Tween 20 in PBS). Cells were washed three times in PBS and stained for 30 min with secondary antibodies from Molecular Probes (goat anti-rabbit AF488 1/1,000+goat anti-mouse AF594 1/1,000). For analysis of 53BP1 NBs or CENPA detection, cells were directly fixed in 4% paraformaldehyde and permeabilized for 10 min in PBS supplemented with 0.5% Triton x100. Cells were immunostained with rabbit anti-53BP1 (1/300)+mouse anti-cyclin A (1/200) or with mouse anti-CENPA (1/500), all from Abcam (#ab21083, #ab16726, #ab13939). For analysis of RPA foci, cells were pulse-labelled with 10 μM EdU for 15 min, extracted with CSK100 and fixed. EdU was detected with the Click-iT EdU Alexa Fluor 488 Imaging kit (Molecular Probes), according to the manufacturer's instructions. Cells were then stained for RPA32 (rabbit anti-RPA32, Bethyl #A300-244A, 1/2,000, detected with goat anti-rabbit AF594). Coverslips were mounted in fluorescent mounting medium (DAKO) supplemented with DAPI. Images were acquired on an Axio Imager Z1 microscope using the Axio Vision software (Zeiss). Intensity was quantified with ImageJ software.

### *In situ* proximity ligation assay

Cells were pulse-labelled with 10 μM EdU for 5 min before pre-extraction and fixation as described above. PLA with nascent DNA was described elsewhere^69^. Briefly, cells were blocked with 3% BSA in PBS. Biotin-azide was conjugated to EdU by click chemistry and cells were incubated with primary antibodies against polη and biotin (rabbit anti-polη 1/300, Santa Cruz H300, and mouse anti-biotin 1/6,000, Jackson ImmunoResearch #200-002-211). PLA and EdU counterstaining were performed according to the manufacturer's instructions using the Duolink *In Situ* Red kit (Sigma) and goat anti-mouse Alexa Fluor 488 antibody.

### *In vitro* transcription/translation of human polη and TLS assay

*In vitro* transcription/translation of full-length WT or mutant polη was performed using a TNT-coupled reticulocyte lysate system (Promega) according to the manufacturer's instructions. The expression vector encoding polη was added to the reaction mixture and incubated for 90 min at 30 °C in the presence of [^35^S] methionine. The catalytic activity of the DNA polymerase was analysed by primer extension on a circular single-stranded template (pUC118) and separation of the labelled products on a 20% polyacrylamide-7 M urea denaturing gel. Construction of single-stranded plasmids containing a single unique TT-CPD (pUC-CDP.ss) has been extensively described^70^. Primer extension analysis was performed as previously described^71^ using a XP30RO cell extract supplemented with an equal amount of WT or mutated polη. Briefly, the reaction mixture (6.25 μl) containing 10 fmoles of primed monomodified DNA and 20 μg of proteins was incubated 20 min at 37 °C in 50 mM Hepes-KOH (pH 7.8), 7 mM MgCl2, 1 mM DTT, 4 mM ATP, 500 μM of dNTPs, 40 mM creatine phosphate, 100 mg per ml creatine kinase. The reaction was stopped by adding an equal volume of proteinase K-SDS (4 mg ml^−1^—2%) and incubated for 30 min at 37 °C. Purified replication products were further digested with EcoRI and PvuII restriction enzymes and analysed by electrophoresis on a polyacrylamide-7 M urea denaturing gel. Radiolabelled products were visualized and quantified after phophorimaging (Typhoon FLA9500) using the ImageQuant TL software.

### Data availability

The data that support the findings of this study are available from the corresponding authors upon request.

## Additional information

**How to cite this article:** Despras, E. *et al*. Rad18-dependent SUMOylation of human specialized DNA polymerase eta is required to prevent under-replicated DNA. *Nat. Commun.* **7,** 13326 doi: 10.1038/ncomms13326 (2016).

**Publisher's note:** Springer Nature remains neutral with regard to jurisdictional claims in published maps and institutional affiliations.

## Supplementary Material

Supplementary Information Supplementary Figures 1-11 and Supplementary References.
