# Supplementary material for: Rad18-dependent SUMOylation of human specialized DNA polymerase eta is required to prevent under-replicated DNA
Source: Nat Commun. 2016 Nov 4;7:13326. doi: 10.1038/ncomms13326 (PMC5097173; doi:10.1038/ncomms13326)
Supplement: Supplementary Information — Supplementary Figures 1-11 and Supplementary References. [file ncomms13326-s1.pdf]

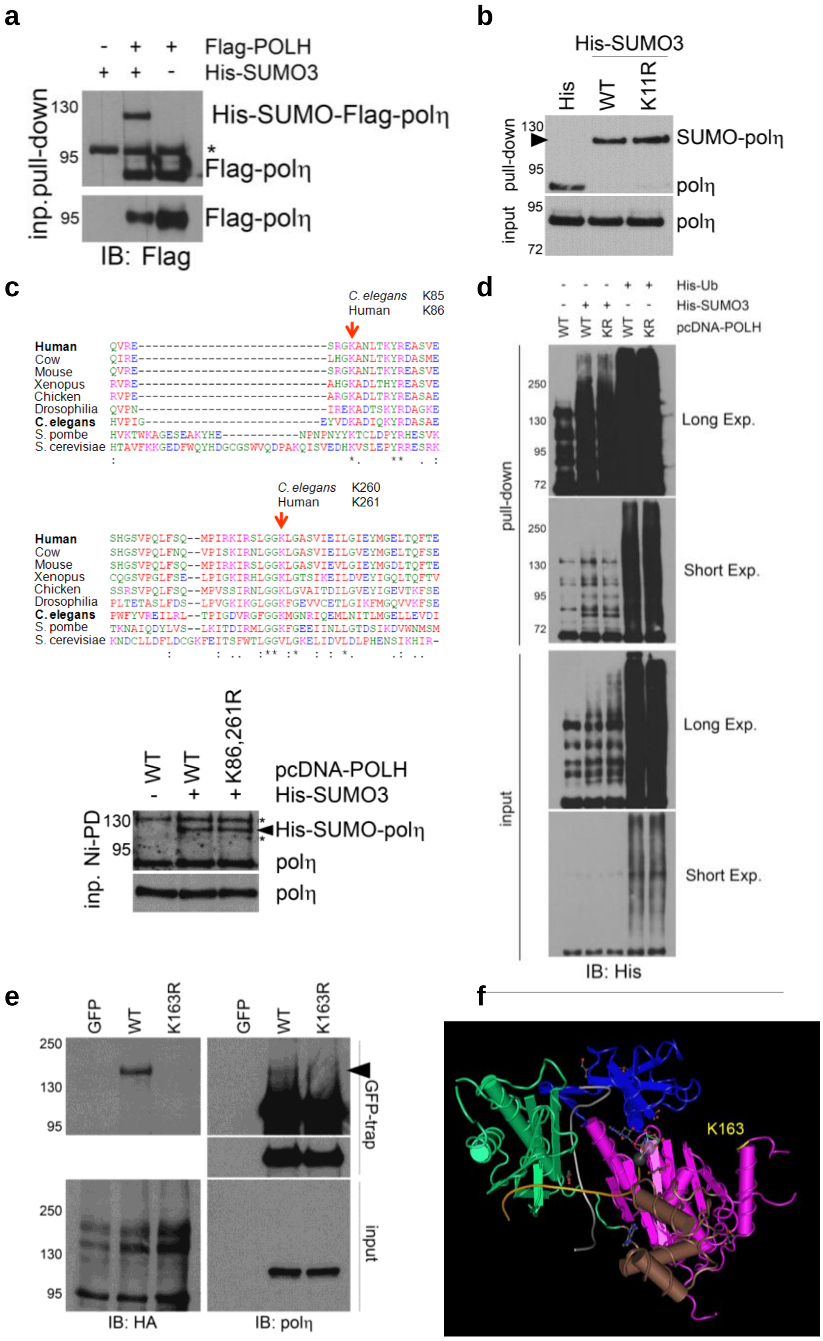

Supplementary Figure 1 (related to Figure 2): **Human polη is SUMOylated *in cellulo*.** (**a**) 293FT cells were co-transfected with plasmids coding for His-tagged SUMO3 (His-SUMO3) and Flag-tagged polη (Flag-POLH). Cells were lysed 24 h after transfection under denaturing conditions. SUMOylated proteins were enriched on Ni beads and analysed by western blot. *: unspecific band. (**b**) SUMOylation of polη was analysed after transfection of His-SUMO3 either WT or mutated on the K11 residue (K11R) by Ni pull-down. (**c**) Upper panel: sequence alignment of polη homologues in various species. Red arrows indicate the two lysines SUMOylated in *C. elegans* polh-1 (K85 and K260)[1] and conserved in human polη (K86 and K261). Lower panel: K86 and K261 were mutated to arginine in human polη and the SUMOylation of the double mutant was analysed by denaturing Ni pull-down after transient expression in XP30RO (XPV) cells together with His-SUMO3. (**d**) His immunoblotting of the experiment presented in Fig. 2c. (**e**) 293FT cells were co-transfected with WT or K163R GFP-polη and HA-SUMO2. GFP-polη was purified on GFP-trap beads extensively washed in stringent denaturing buffer. Samples were analysed by western blot with anti-polη and anti-HA antibodies. (**f**) K163 residue (in yellow) was positioned on the structure of human polη catalytic domain in complex with normal DNA and incoming nucleotide[2] (pink: palm, brown: thumb, blue: fingers, green: little finger, grey: DNA template, orange: DNA primer) using Cn3D software. (http://www.ncbi.nlm.nih.gov/Structure/CN3D/cn3d.shtml)

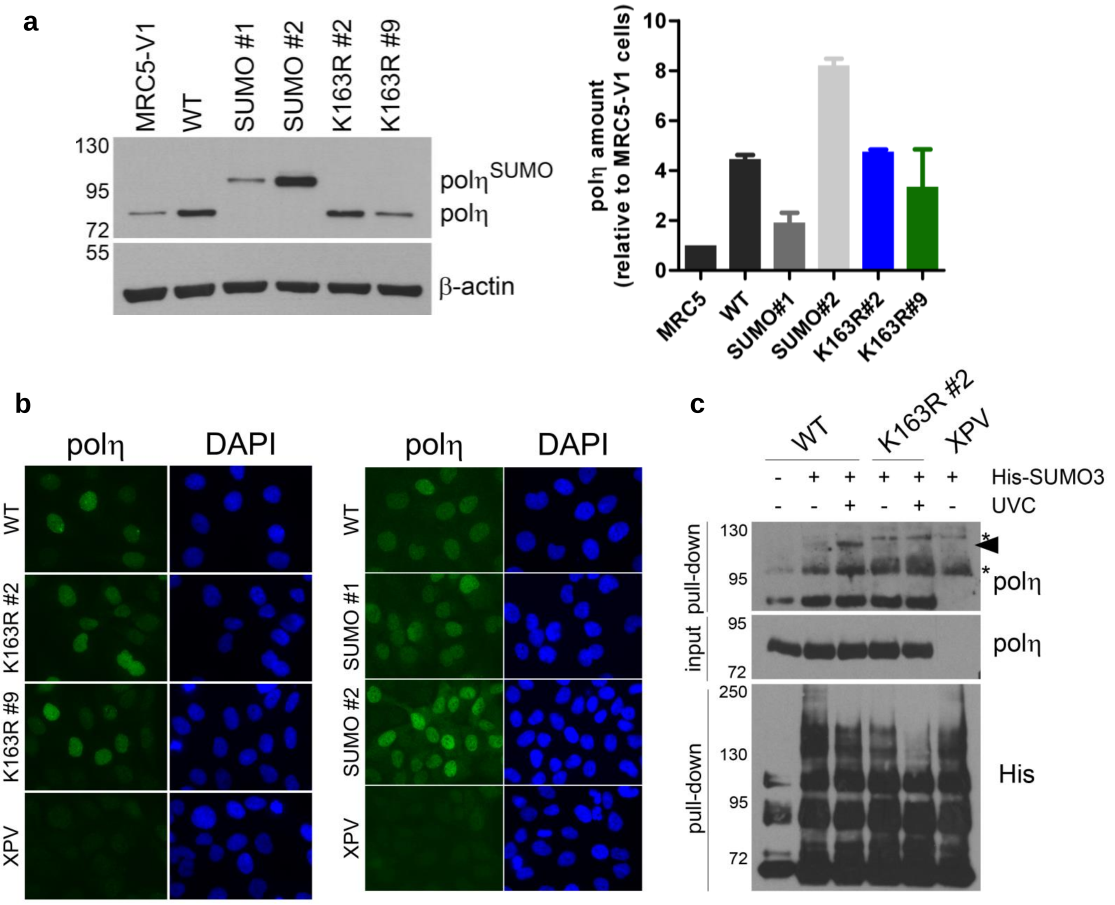

Supplementary Figure 2 (related to Figure 4): **Establishment of cell lines stably expressing polη^{K163R} and polη^{SUMO}.** XP30RO (XPV) cells were transfected with a plasmid coding for K163R polη (polη^{K163R}) or constitutively SUMOylated polη (polη^{SUMO}). Clones from two independent transfections were selected and characterized for further experiments. SUMO #1 and #2 express the SUMOa and SUMOb constructs, respectively (see Methods). Complementation of XP30RO cells with polη^{WT} has already been described[3]. (**a**) Western blot analysis of polη amounts in normal MRC5-V1 fibroblasts and in XPV cells reconstituted with polη^{WT}, polη^{SUMO} and polη^{K163R} (left panel). Quantification was done using ImageJ software (right panel). β-actin served as a loading control. The amounts of polη were expressed as a ratio to MRC5-V1 cells. Data are the mean ± s.d. of two independent experiments. (**b**) The nuclear localisation of the various polη mutants was confirmed by immunofluorescence on fixed total cells. (**c**) The indicated cell lines were transfected with His-SUMO3. 24 h after transfection, cells were irradiated or not at 20 J.m^{-2} and incubated for 6 h before Ni pull-down. *: unspecific band.

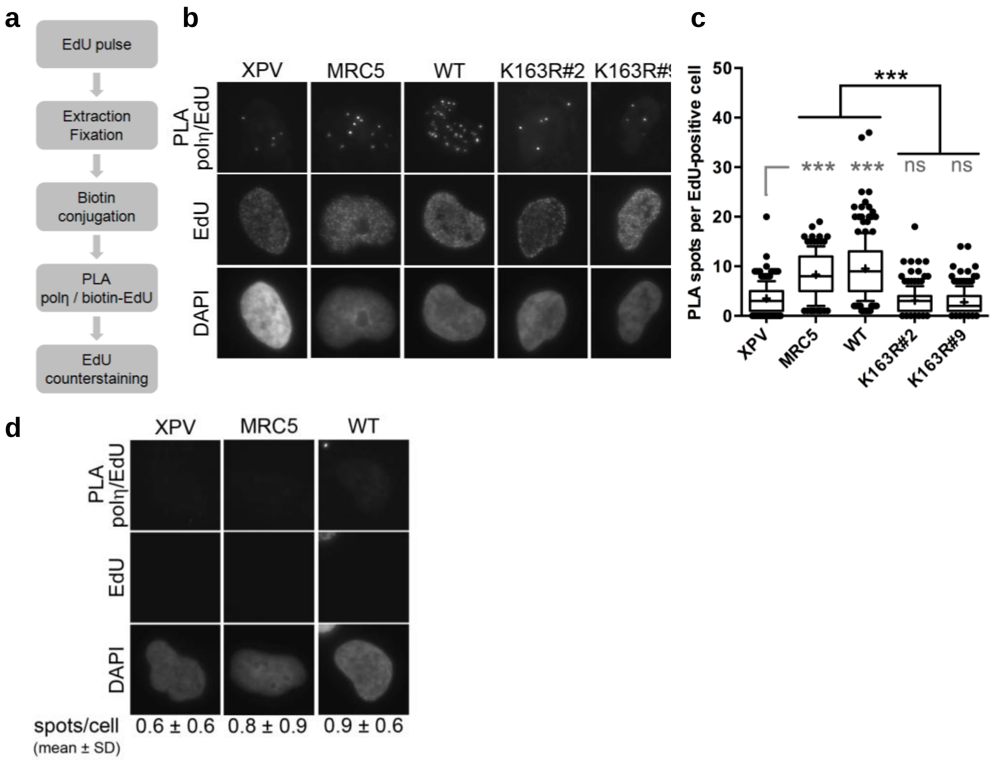

Supplementary Figure 3 (related to Figure 4): **polη^{K163R} is deficient in recruitment to nascent DNA during unperturbed S phase.** (**a**) Scheme for the in situ Proximity Ligation Assay (PLA) between polη and nascent DNA. Cells were pulse-labelled with EdU for 5 min prior to pre-extraction and fixation. Biotin-azide was conjugated to EdU by click chemistry. PLA was performed between polη and EdU-biotin and total EdU-biotin was further counterstained to unravel S phase cells. (**b**) Representative images of EdU-positive XPV, MRC5-V1, polη^{WT} and polη^{K163R} cells (magnification x63). (**c**) The distribution of the number of PLA spots per EdU-positive cells is presented in a box-plot with 10-90 percentile whiskers (n>160, ns: not significant, ***: p<0.001, Mann-Whitney test). (**d**) Representative images of EdU-negative XPV, MRC5-V1 and polη^{WT} cells (magnification x63) with the mean number ± s.d. of PLA spots (n>100).

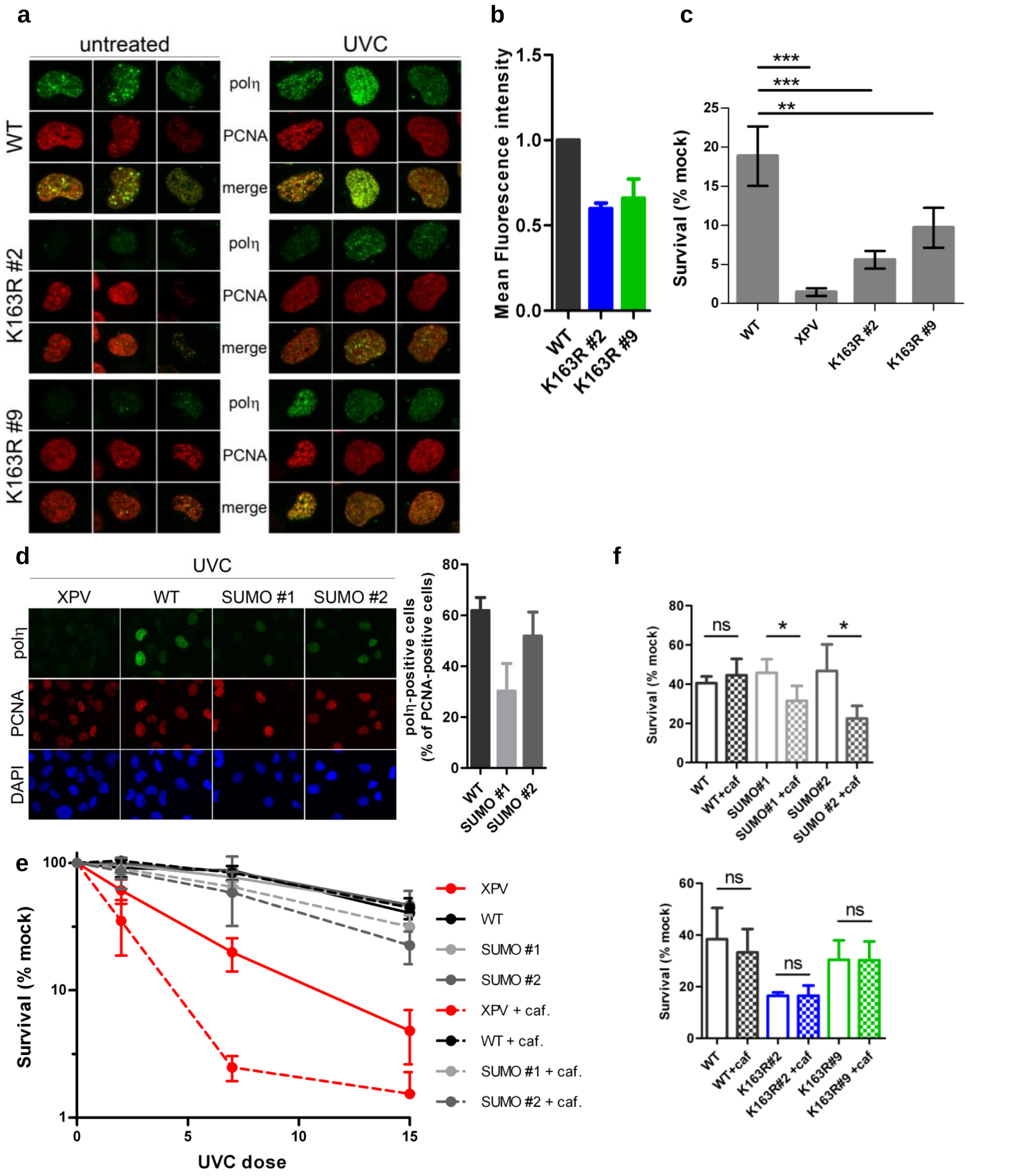

Supplementary Figure 4 (related to Figures 4 and 5): **polη^{K163R} is impaired in localization to replication foci but can complement the UV sensitivity of XPV cells.** (**a**) Zoom on representative staining patterns obtained during the experiments shown in Fig. 4a and 5c. (**b**) Fluorescence intensity of polη immunostaining was quantified in polη-positive cells after UV using ImageJ software. Mean intensity of polη^{K163R} signal was expressed as the ratio to mean intensity of polη^{WT} signal (mean ± s.d. of three independent experiments). (**c**) XPV, polη^{WT} and polη^{K163R} cells were irradiated at 20 J.m^{-2} and grown for 72 h before cell counting in presence of trypan blue (mean ± s.d. of four independent experiments). (**d**) XPV, polη^{WT} and polη^{SUMO} cells were treated as in Fig. 5c. (**e**) XPV, polη^{WT} and polη^{SUMO} cells were treated as in Fig. 5e. Values are the mean ± s.d. of four independent experiments. (**f**) Surviving fractions after 15 J.m^{-2} from experiments described in (e) (upper panel) and Fig. 5e (lower panel). ns: not significant, *: $p<0.05$ (t-test).

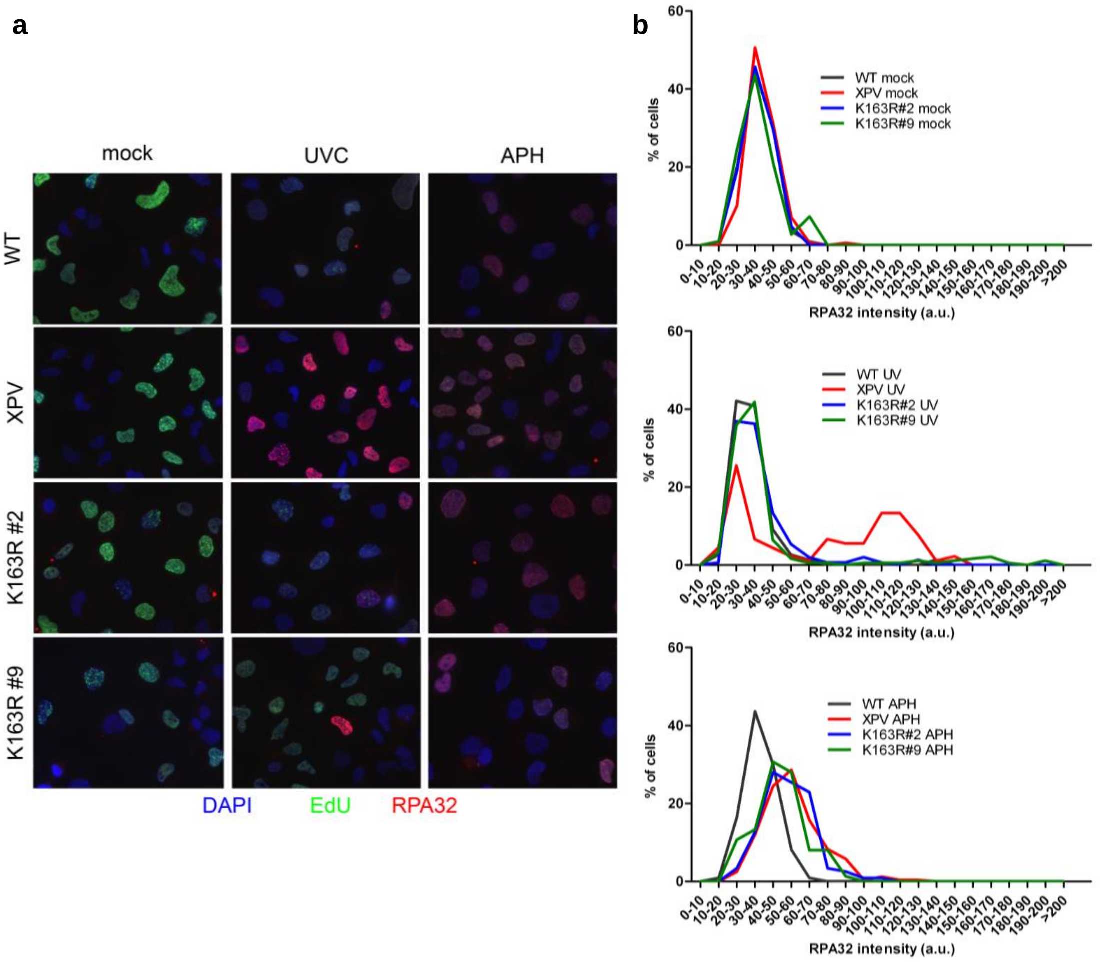

Supplementary Figure 5 (related to Figures 5 and 6): **A low dose of APH, but not UVC, leads to single-stranded DNA accumulation during S phase in polη^{K163R} cells.** XPV, polη^{WT} and polη^{K163R} cells were treated with 7 $J.m^{-2}$ for 6 h or 0.3 µM APH for 24 h. S phase cells were labelled with EdU. Soluble proteins were extracted prior to fixation and detection of EdU (in green) and RPA32 protein (in red). Mean RPA32 intensity was quantified in EdU-positive cells using ImageJ software. (**a**) Representative images (magnification x63). (**b**) Distribution of mean RPA32 intensity in S phase cells (arbitrary unit).

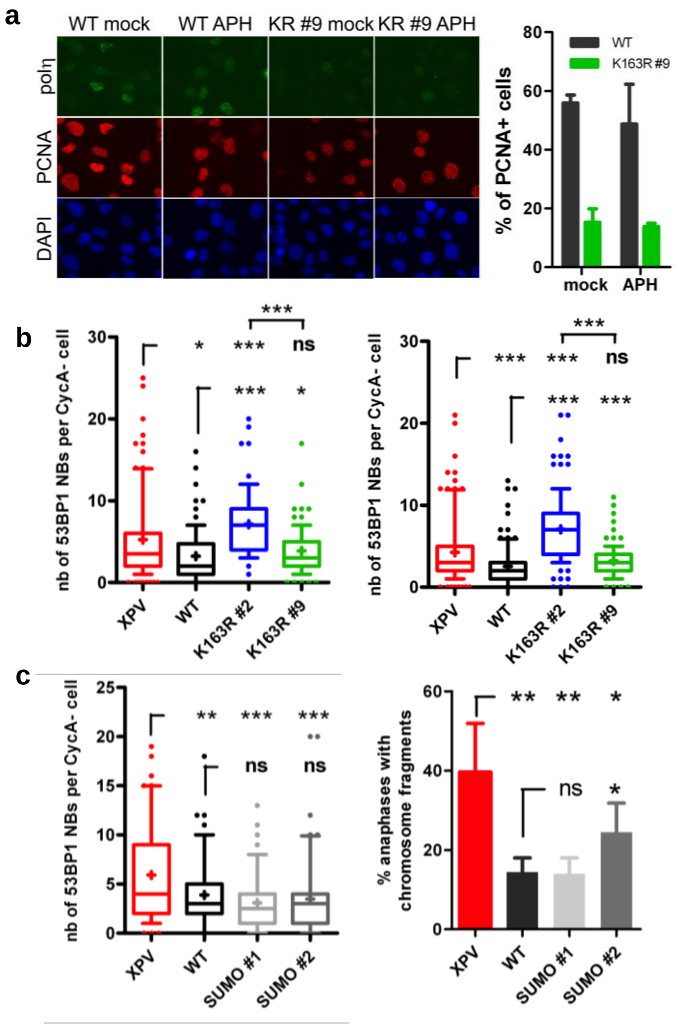

Supplementary Figure 6 (related to Figure 6): **polη SUMOylation is required in response to mild replication stress.** (**a**) XPV cells stably expressing polη^{WT} or polη^{K163R} (clone #9) were treated with 0.3 μM APH for 24 h and processed as in Fig. 4a. Representative images are shown on the left panel (magnification x63). The proportion of PCNA-positive cells presenting polη foci is shown in the right panel (mean ± s.d. of two independent experiments). (**b**) Analysis of 53BP1 NBs as in Fig. 6c,d in two other independent experiments (n=100, ns: not significant, *: p<0.05, ***: p<0.001, Mann-Whitney test). (**c**) Analysis of 53BP1 NBs (left panel, n=100, ns: not significant, **: p<0.01, ***: p<0.001, Mann-Whitney test) and lagging chromosome fragments in anaphase (right panel, mean ± s.d. of five independent experiments, n=50 per experiment, ns: not significant, *: p<0.05, **: p<0.01, t-test) in polη^{SUMO} cells treated with 0.3 μM APH.

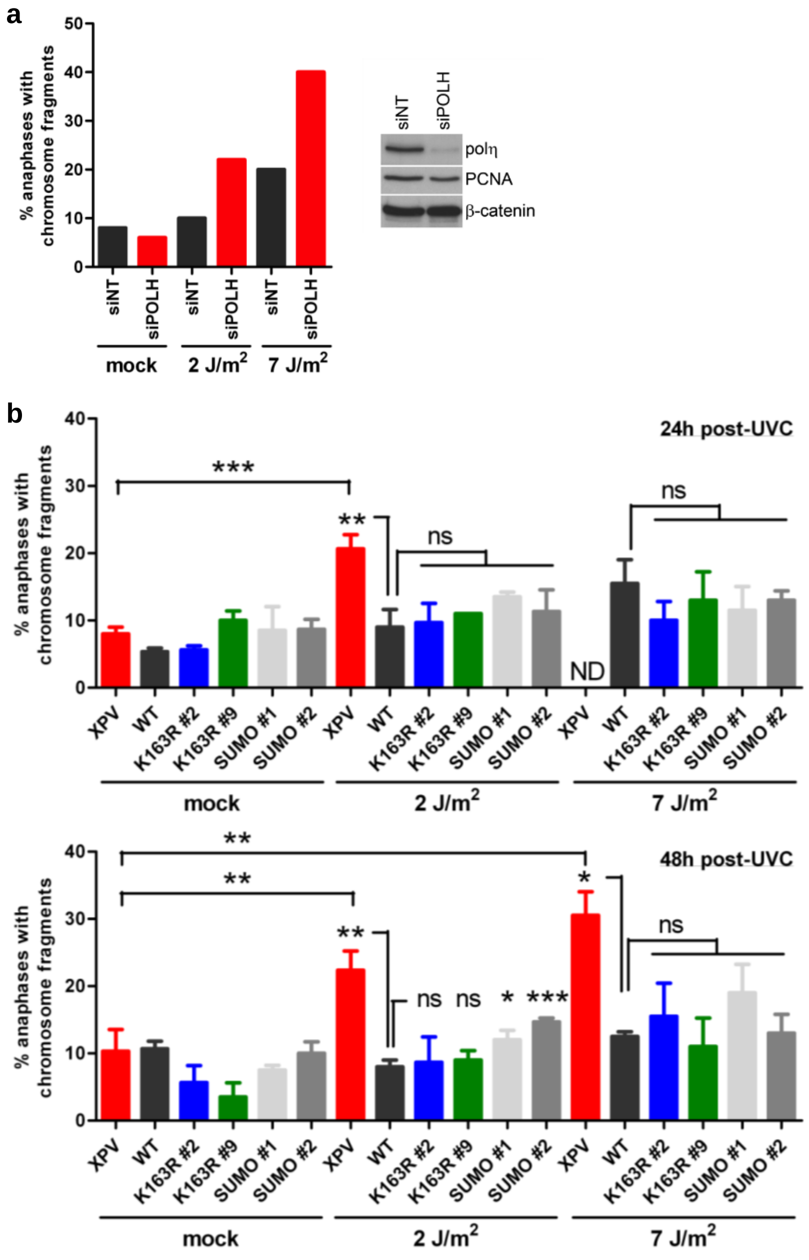

Supplementary Figure 7 (related to Figure 6): **Polη deficiency leads to increased aberrant anaphases after UVC, which can be rescued by polη^{K163R} expression.** The proportion of anaphases with lagging chromosome fragments was assessed after irradiation at the indicated UVC doses (**a**) in polη-depleted MRC5-V1 cells 24 h after irradiation and (**b**) in XPV cells and XPV cells stably expressing polη^{WT}, polη^{K163R} or polη^{SUMO} 24 h and 48 h after irradiation (mean ± s.d., 3 independent experiments for XPV, polη^{WT}, polη^{K163R} #2 and polη^{SUMO} #2 after 2 J/m^2, 2 independent experiments for polη^{K163R} #9 and polη^{SUMO} #1 and after 7 J/m^2, n=100 per experiment, ns: not significant, *: $p<0.05$, **: $p<0.01$, ***: $p<0.001$, t-test). Note that the percentage of aberrant anaphases was not assessed in XPV cells 24 h after 7 J/m^2 (ND) because cells are mostly accumulated in S phase in this condition[4].

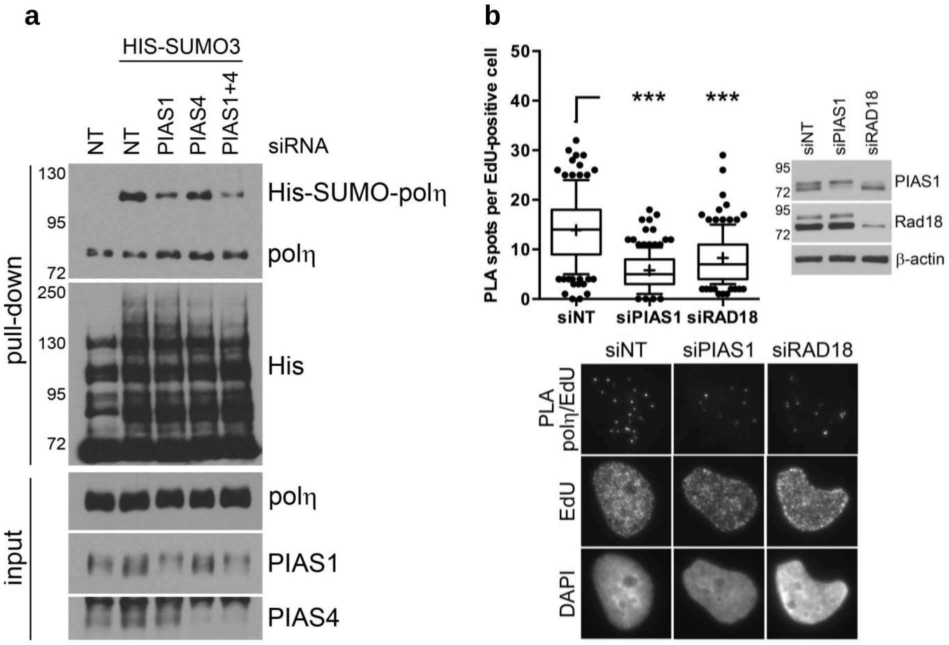

Supplementary Figure 8 (related to Figure 7): **Polη is SUMOylated in a PIAS1-dependent manner.** (**a**) 293FT cells were transfected with the indicated siRNAs 24 h before co-transfection of plasmids expressing polη and His-SUMO3. Polη SUMOylation was analysed as in Fig. 2. (**b**) MRC5-V1 cells were transfected with the indicated siRNAs 72 h before EdU pulse and fixation. PLA between EdU and polη was performed as described in Supplementary Fig. 3. Upper left panel: distribution of the number of PLA spots per EdU-positive cells (n>130, ***: p<0.001, Mann-Whitney test). Upper right panel: western blot showing the efficiency of the siRNAs. Lower panel: representative images (magnification x63).

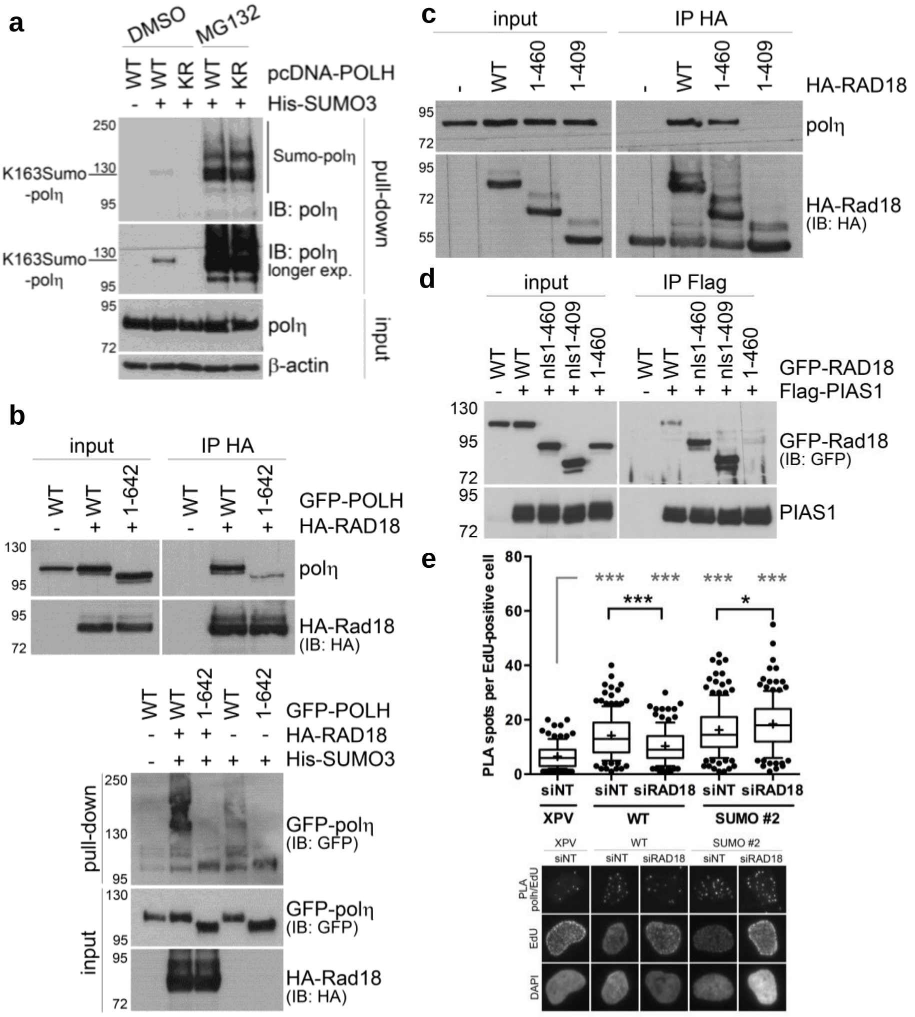

Supplementary Figure 9 (related to Figure 8): **Rad18 is required for efficient interaction between polη and PIAS1 and subsequent polη SUMOylation.** (**a**) 293FT cells were co-transfected with pcDNA-POLH WT or K163R (KR) and His-SUMO3. Cells were treated for 3 h with 10 μM MG132 prior to denaturing Ni pull-down as in Fig. 2 and immunoblotting. (**b**) Upper panel: 293FT cells were transfected with GFP-polη^{WT} or C-terminal truncated GFP-polη^{1-642} and HA-Rad18. Rad18 was immunoprecipitated with an anti-HA antibody and co-immunoprecipitated polη levels were determined by western blot. Lower panel: 293FT cells were transfected with the same plasmids and His-SUMO3 24 h before Ni pull-down and immunoblotting using the indicated antibodies. (**c**) 293FT cells were co-transfected with plasmids expressing polη and HA-Rad18^{WT}, HA-Rad18$^{1-460}$ or HA-Rad18$^{1-409}$. Immunoprecipitation was performed with an anti-HA antibody and immunoprecipitated proteins were analysed by western blot. (**d**) 293FT cells were co-transfected with plasmids expressing Flag-PIAS1 and the indicated GFP-Rad18 constructs. Immunoprecipitation was performed with an anti-Flag antibody and further analysed by western blot. (**e**) PLA between nascent DNA and polη was performed in polη^{WT} and polη^{SUMO} cells depleted for Rad18 as described in Supplementary Fig. 3. The distribution of the number of PLA spots per EdU-positive cells is shown in the upper panel (n>130, *: $p<0.05$, ***: $p<0.001$, Mann-Whitney test). Lower panel: representative images (magnification x63).

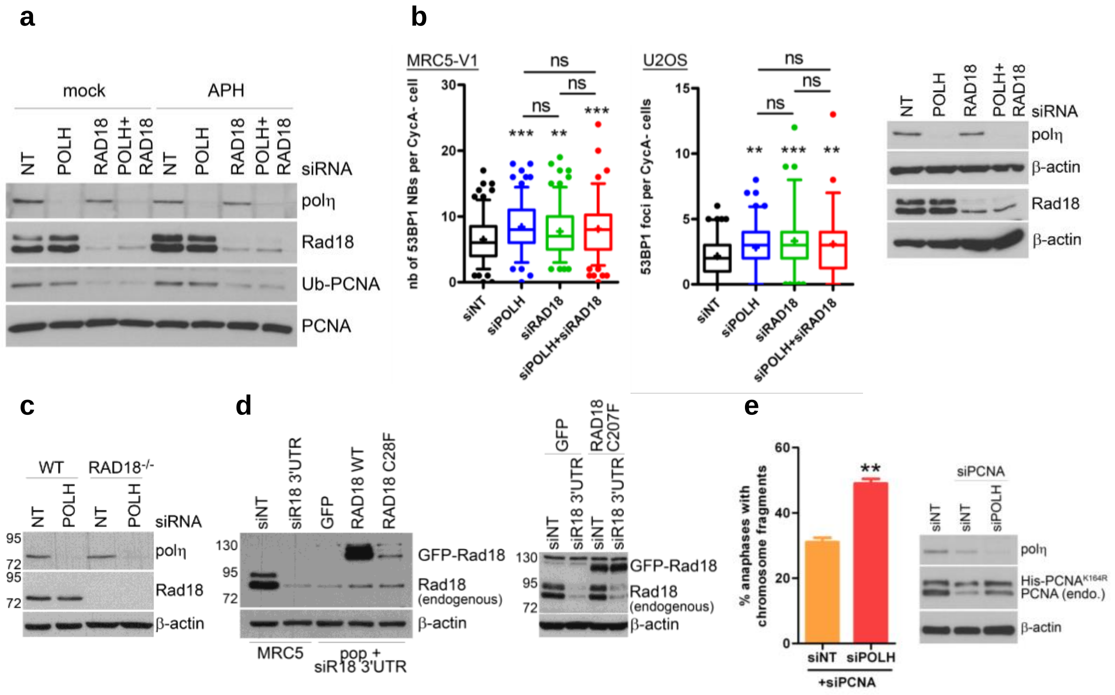

Supplementary Figure 10 (related to Figure 9): **Polη and Rad18 act in the same pathway in response to APH.** (**a**) Analysis of siRNAs efficiency in MRC5-V1 cells (refers to Fig. 9a). (**b**) MRC5-V1 and U2OS cells were transfected with siRNAs directed against polη and/or Rad18 mRNAs 48 h before treatment with 0.15 and 0.3 µM APH, respectively, for 24 h. Cells were fixed and stained for 53BP1 and Cyclin A. The number of 53BP1 NBs was assessed in at 100 cyclin A-negative cells. Left panel: MRC5-V1 cells. Middle panel: U2OS cells. Right panel: analysis of siRNA efficiency in U2OS cells. ns: not significant, **: p<0.01, ***: p<0.001 (Mann-Whitney test). (**c**) Analysis of siRNAs efficiency in HCT116 cells (refers to Fig. 9b). (**d**) Analysis of siRad18 3'UTR efficiency in MRC5-V1 cells and GFP-Rad18 expressing populations (refers to Fig. 9c,d). (**e**) The proportion of anaphases with lagging chromosome fragments was assessed in MRC5 cells stably expressing His-PCNA^{K164R} after depletion of endogenous PCNA and polη and treatment with 0.15 µM APH for 24 h (left panel). Values are the mean ± s.d. of 2 independent experiments. **: p<0.01 (t-test). siRNAs efficiency was confirmed by western blot (right panel).

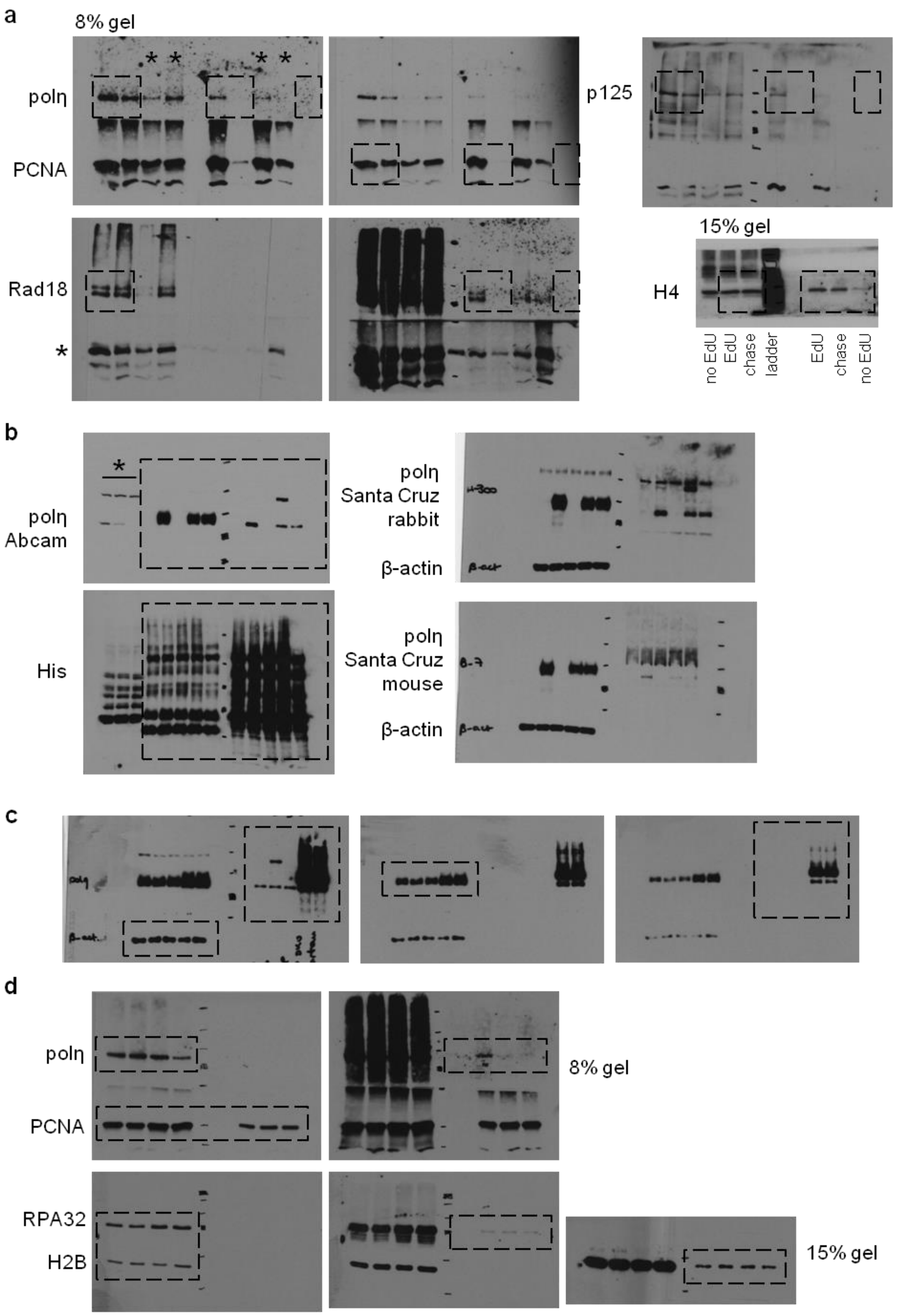
a
8% gel
polη
PCNA
p125
Rad18
*
15% gel
H4
no EdU
EdU
chase
ladder
EdU
chase
no EdU
b
polη
Abcam
His
polη
Santa Cruz
rabbit
β-actin
polη
Santa Cruz
mouse
β-actin
c
d
polη
PCNA
8% gel
RPA32
H2B
15% gel

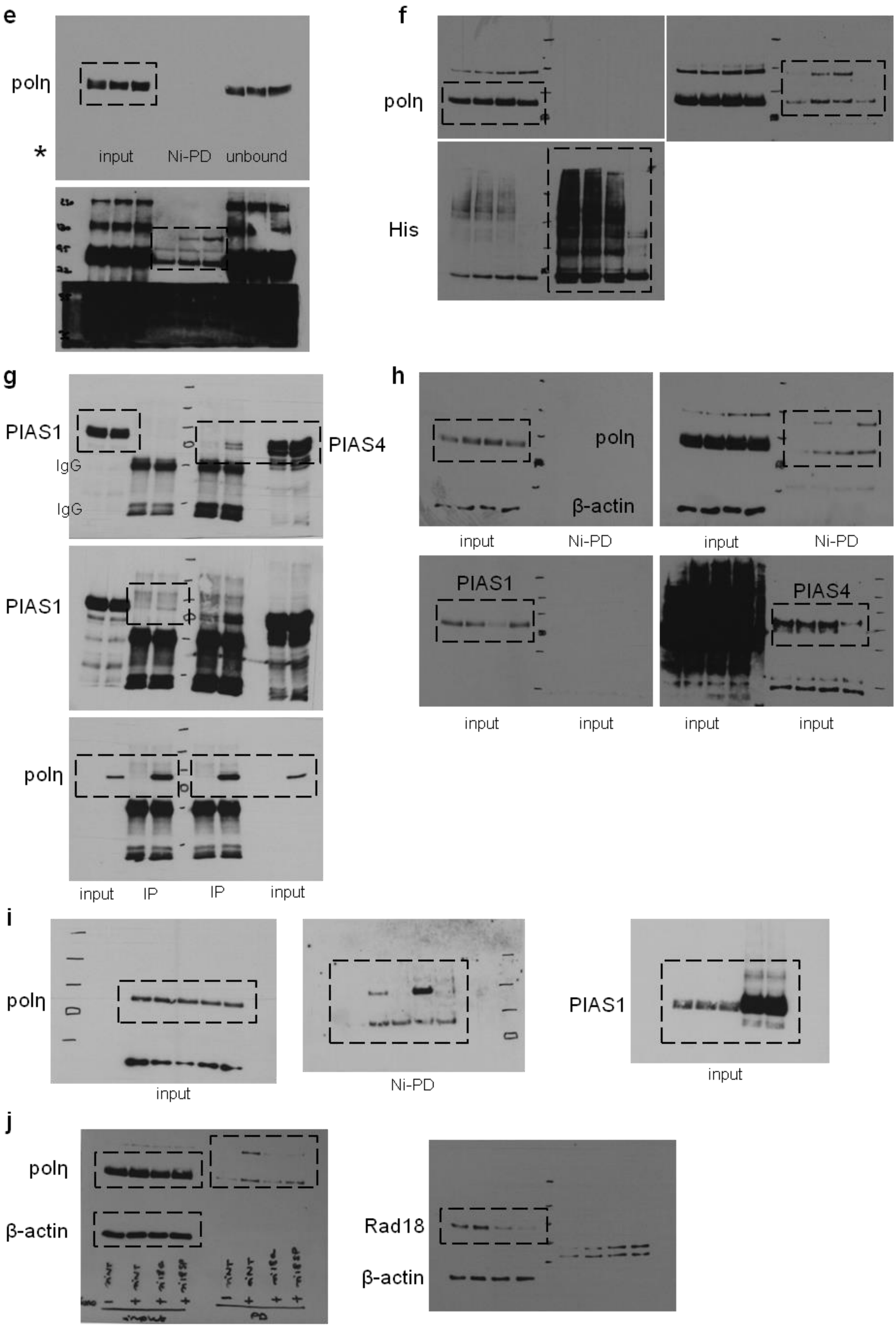
e
polη
*
input
Ni-PD
unbound
f
polη
His
g
PIAS1
IgG
IgG
PIAS4
PIAS1
polη
input
IP
IP
input
h
polη
β-actin
input
Ni-PD
input
Ni-PD
PIAS1
PIAS4
input
input
input
input
i
polη
input
Ni-PD
PIAS1
input
j
polη
β-actin
Rad18
β-actin

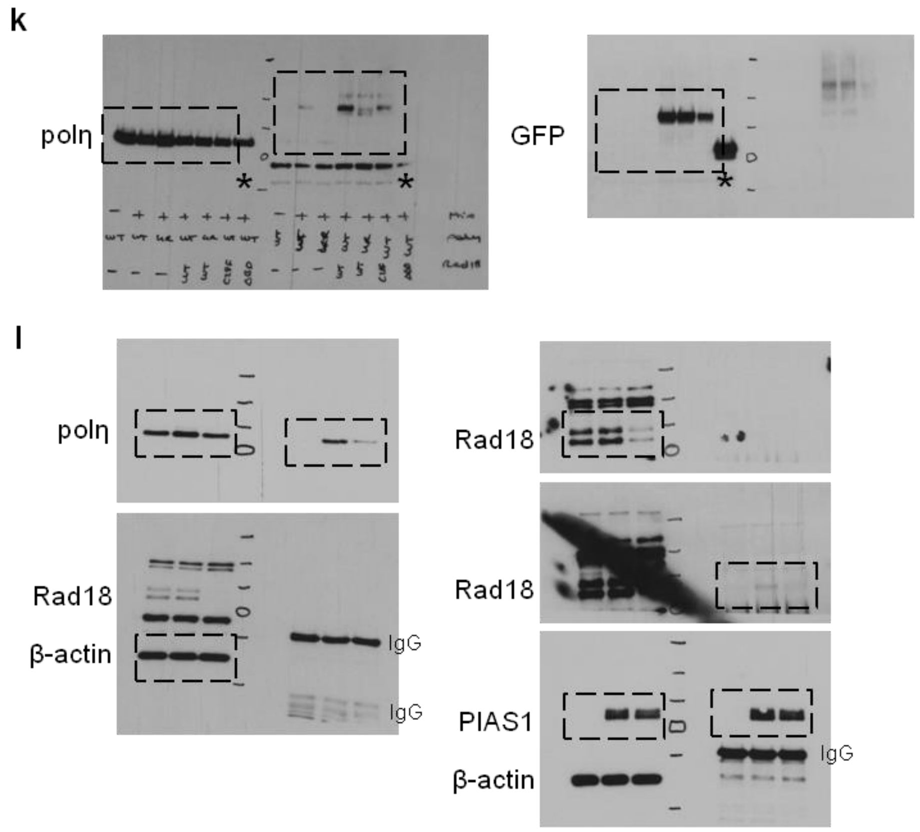

Supplementary Figure 11: **Non cropped images of the most relevant western blots.** (**a**) Fig. 1b. (**b**) Fig. 2a. (**c**) Fig. 2c. (**d**) Fig. 4e. (**e**) Fig. 5a. (**f**) Fig. 5b. (**g**) Fig. 7a. (**h**) Fig. 7c (**i**) Fig. 7d. (**j**) Fig. 8a. (**k**) Fig. 8c. (**l**) Fig. 8h *: samples or proteins not related to the present study.

SUPPLEMENTARY REFERENCES

1. Kim, S.H. & Michael, W.M. Regulated proteolysis of DNA polymerase eta during the DNA-damage response in C. elegans. *Mol Cell* **32**, 757-766 (2008).
2. Biertumpfel, C. *et al.* Structure and mechanism of human DNA polymerase eta. *Nature* **465**, 1044-1048 (2010).
3. Stary, A., Kannouche, P., Lehmann, A.R. & Sarasin, A. Role of DNA polymerase eta in the UV mutation spectrum in human cells. *J Biol Chem* **278**, 18767-18775 (2003).
4. Despras, E., Daboussi, F., Hyrien, O., Marheineke, K. & Kannouche, P.L. ATR/Chk1 pathway is essential for resumption of DNA synthesis and cell survival in UV-irradiated XP variant cells. *Hum Mol Genet* **19**, 1690-1701 (2010).
